# Albumin-Mediated Drug Uptake by Organic Anion Transporter 1/3 Is Real: Implications for the Prediction of Active Renal Secretion Clearance

**DOI:** 10.1021/acs.molpharmaceut.4c00504

**Published:** 2024-08-21

**Authors:** Shawn Pei Feng Tan, Annika Tillmann, Susan J. Murby, Amin Rostami-Hodjegan, Daniel Scotcher, Aleksandra Galetin

**Affiliations:** † Centre for Applied Pharmacokinetic Research, School of Health Sciences, University of Manchester, Manchester M13 9PL, U.K.; ‡ Certara Predictive Technologies (CPT), Certara Inc., 1 Concourse Way, Sheffield S1 2BJ, U.K.

**Keywords:** *in vitro*-to-*in vivo* extrapolation, drug transporters, renal clearance, organic anion transporter 1/3

## Abstract

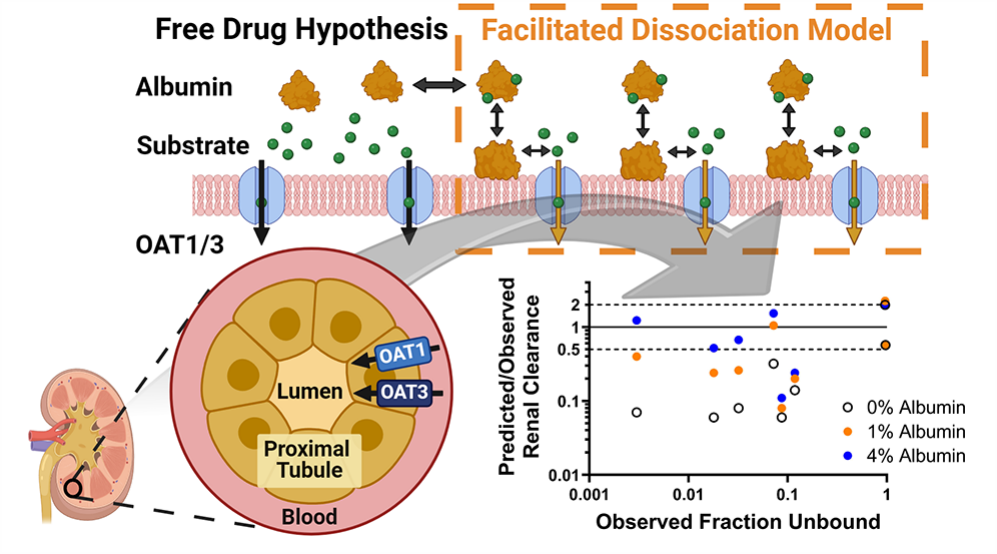

Modulation of the transport-mediated active uptake by human serum albumin (HSA) for highly protein-bound substrates has been reported and improved the *in vitro*-to-*in vivo* extrapolation (IVIVE) of hepatic clearance. However, evidence for the relevance of such a phenomenon in the case of renal transporters is sparse. In this study, transport of renal organic anion transporter 1 or 3 (OAT1/3) substrates into conditionally immortalized proximal tubular epithelial cells transduced with OAT1/3 was measured in the presence and absence of 1 and 4% HSA while keeping the unbound substrate concentration constant (based on measured fraction unbound, *f*_u,inc_). In the presence of 4% HSA, the unbound intrinsic active uptake clearance (CL_int,u,active_) of six highly protein-bound substrates increased substantially relative to the HSA-free control (3.5- to 122-fold for the OAT1 CL_int,u,active_, and up to 28-fold for the OAT3 CL_int,u,active_). The albumin-mediated uptake effect (fold increase in CL_int,u,active_) was more pronounced with highly bound substrates compared to no effect seen for weakly protein-bound substrates adefovir (OAT1-specific) and oseltamivir carboxylate (OAT3-specific). The relationship between OAT1/3 CL_int,u,active_ and *f*_u,inc_ agreed with the facilitated-dissociation model; a relationship was established between the albumin-mediated fold change in CL_int_,_u,active_ and *f*_u,inc_ for both the OAT1 and OAT3, with implications for IVIVE modeling. The relative activity factor and the relative expression factor based on global proteomic quantification of *in vitro* OAT1/3 expression were applied for IVIVE of renal clearance. The inclusion of HSA improved the bottom-up prediction of the level of OAT1/3-mediated secretion and renal clearance (CL_sec_ and CL_r_), in contrast to the underprediction observed with the control (HSA-free) scenario. For the first time, this study confirmed the presence of the albumin-mediated uptake effect with renal OAT1/3 transporters; the extent of the effect was more pronounced for highly protein-bound substrates. We recommend the inclusion of HSA in routine *in vitro* OAT1/3 assays due to considerable improvements in the IVIVE of CL_sec_ and CL_r_.

## Introduction

There has been a resurgence of interest in the “albumin-mediated uptake effect” with the aim to bridge the significant underprediction of hepatic transporter-mediated clearance during early drug development.^1,2^ Addition of albumin or plasma to hepatocyte assays^1−4^ and immortalized cells expressing hepatic organic anion transporting polypeptide (OATP1B1/3)^5−8^ led to substantial alteration of *in vitro* transporter kinetic parameters of OATP1B1/3 substrates beyond that expected from the free-drug hypothesis.^9^ Human serum albumin (HSA) is the major plasma protein (approximately 35 to 50 g/L),^10^ and many endogenous substances such as lipids, steroids, hormones, and xenobiotics bind to this protein.^11,12^ The effect of albumin on transporter substrates was first described in studies with sulfobromophthalein.^13^ Since then, several studies have observed the albumin-mediated effect with liver models^14−17^ and rabbit proximal tubules.^18^ Clinical evidence of this phenomenon is limited, but studies have trialed an intravenous dose of furosemide with albumin,^19,20^ leading to greater furosemide induced diuresis.^21^

Thus far, published studies investigating the albumin-mediated uptake effect with hepatic OATP1B1/3 transporters have employed a variety of experimental conditions.^1,9^ These studies differed in the choice of plasma protein (human/bovine serum albumin or plasma), the concentration of albumin used (0.125 to 10%), cell type (human/rat hepatocytes or immortalized cell lines), the concentration of the unbound drug in albumin and albumin-free condition, and assay format (plated cells or oil-spin hepatocytes). As a consequence, varying extents of the albumin-mediated uptake effect on transporter activity have been reported across studies, even for the same OATP1B1/3 substrate.^1^ Nonetheless, it has been consistently shown that highly protein-bound drugs are more significantly affected by the albumin-mediated uptake effect.^1,6,22^ The inclusion of plasma proteins leads to a decrease in the total intrinsic clearance (CL_int_) measured. However, upon normalizing CL_int_ by the extent of drug-protein binding in the experimental condition, the unbound CL_int_ (CL_int,u_) measured in the presence of albumin or plasma increased beyond that of the protein-free control condition.^1−3,6,23^ The albumin-mediated uptake effect was albumin concentration dependent^2^ and was also observed with membrane vesicles.^24^

Several plausible mechanisms behind the albumin-mediated uptake effect have been proposed.^4,9,22,25^ Although no consensus has been met, all proposed mechanisms are distinct from the effect of albumin reported on uridine 5′-diphospho-glucuronosyltransferase.^26−28^ Among these, the facilitated-dissociation model proposed by Tsao et al.^15^ has been strongly supported by experimental transporter data.^2,3,6,22^ In this model, albumin is proposed to interact with a surface receptor on the cell membrane, leading to a conformational change in albumin and subsequent dissociation of any albumin-bound ligand from the albumin–ligand complex.^2,3,22^ This process provides additional unbound ligands (transporter substrate) available for transporter uptake and increases the measured CL_int,u_ beyond that anticipated by the conventional free-drug hypothesis. Consequently, several studies have demonstrated that the inclusion of albumin or plasma in the experimental setup has led to an improvement in the underprediction of total hepatic clearance for OATP1B1/3 substrates.^3,6,29,30^

Renal organic anion transporters 1/3 (OAT1/3) are clinically relevant transporters located on the basolateral membrane of the renal proximal tubules and responsible for the renal secretion of organic anions from the bloodstream into the renal tubule for urinary excretion.^31^ Renal clearance (CL_r_) of substrates for OAT1/3 is also associated with underprediction using either static *in vitro*-to-*in vivo* extrapolation (IVIVE)^32,33^ or dynamic physiologically based pharmacokinetic (PBPK) modeling approaches.^34−36^ This underprediction is apparent despite significant progress made in understanding and considering the relative difference in transporter expression or activity between the *in vitro* system and *in* vivo tissue as a scaling factor.^37,38^ In a recent study from our group, the albumin-mediated uptake of indoxyl sulfate (a uremic solute) was reported with the renal OAT1 in the presence of HSA and chronic kidney disease (CKD)-modified HSA.^39^ During CKD, conformation changes such as post-translational guanidinylation to HSA occur, causing a decrease in albumin-binding capacity.^40^ The use of HSA was the key to recapitulating the decrease in the level of the OAT1-mediated CL_r_ of indoxyl sulfate from healthy to CKD, highlighting complex drug–disease interactions between CKD and active transporters and the role that albumin possibly plays in this interaction. Nevertheless, there is currently no information on the albumin-mediated uptake effect for a broader range of OAT1/3 substrates and the implications of this phenomenon on improving IVIVE of OAT1/3-mediated CL_r_.

This study aimed to evaluate the albumin-mediated uptake effect systematically using eight renally excreted OAT1/3 substrates with varying extents of plasma protein binding and ascertain if the proposed facilitated-dissociation model could describe this phenomenon. The intracellular uptake of the OAT1/3 substrates was measured using a conditionally immortalized proximal tubular epithelial cell line overexpressing OAT1 (ciPTEC-OAT1) or OAT3 (ciPTEC-OAT3) in the presence and absence of 1 and 4% HSA, along with parallel measurements of the extent of albumin binding. Thereafter, the OAT1/3-mediated CL_int,u_ in the presence of HSA was estimated to evaluate the extent of the albumin-mediated uptake effect. Finally, IVIVE of uptake data obtained in the presence of albumin was performed using the relative expression (REF) and relative activity (RAF) scaling factors to understand the implications of albumin inclusion on the bottom-up prediction of *in vivo* CL_r_ and secretion clearance (CL_sec_) for OAT1/3 substrates.

## Experimental Section

### Chemicals and Materials

ciPTEC cells with stable overexpression of either OAT1 or OAT3 (ciPTEC-OAT1 or ciPTEC-OAT3) were obtained from Cell4Pharma (Oss, Netherlands). Dulbecco’s modified Eagle’s medium and Ham’s F-12 without phenol red (DMEM/F-12, 11039047), Dulbecco’s phosphate-buffered saline (DPBS, D1408), fetal bovine serum (A5256801), UltraPure distilled water (10977035), and HEPES (10397023) were purchased from Gibco, ThermoFisher Scientific (Loughborough, U.K.). 4-Pyridoxic acid (P9630), probenecid (P8761), adefovir (SML0240), furosemide (F4381), rosuvastatin calcium (SML1264), gemfibrozil (G9518), human serum albumin (A1887), Hank’s balanced salt solution (HBSS, H8264), insulin (I2643), transferrin (T8158), sodium selenite (214485), epidermal growth factor (SRP3027), hydrocortisone (H0135), tri-iodothyronine (T5516), and penicillin–streptomycin (P0781) were purchased from Sigma-Aldrich (Dorset, U.K.). Bumetanide (CAY14630), rivaroxaban (B1854), and olmesartan (CAY23412) were purchased from Cambridge Bioscience (Cambridge, U.K.). Oseltamivir carboxylate (A3689) was obtained from Stratech (Cambridge, U.K.). Tissue culture-treated 24-well plates (3526) and T75 flasks (430641U) were purchased from Corning (Flintshire, U.K.). A 96-well equilibrium dialysis device (1007), Teflon block inserts (1003), and 12–14 kDa cellulose membranes (1101) were purchased from HTDialysis, LLC (Gales Ferry, Connecticut).

### ciPTEC Cell Culture

ciPTEC-OAT1 and ciPTEC-OAT3 were cultured following the manufacturer’s recommended protocol.^41^ Briefly, ciPTECs were cultured in T75 flasks with complete ciPTEC culture media and incubated at 33 °C and 5% CO_2_ under a humidified atmosphere. Complete ciPTEC culture media were prepared using DMEM/F-12 without phenol red, supplemented with 5 μg/mL insulin, 5 μg/mL transferrin, 5 ng/mL sodium selenite, 10 ng/mL epidermal growth factor, 36 ng/mL hydrocortisone, 0.04 ng/mL tri-iodothyronine, 1% penicillin–streptomycin, and 10% fetal bovine serum. Cells were cultured to 80–90% confluency and passaged when necessary (between passage number 47 and 60). For uptake transporter experiments, ciPTECs were seeded on 24-well plates in penicillin–streptomycin-free complete ciPTEC culture media at a density of 120,000 cells (ciPTEC-OAT1) or 150,000 cells per well (ciPTEC-OAT3). ciPTECs were allowed to proliferate and adhere to the well plate at 33 °C for 1 day, followed by culturing at 37 °C for 7 days to allow maturation into a confluent cell monolayer. Complete ciPTEC culture medium was replaced every 2–3 days.

### OAT1/3 Uptake Studies

To investigate the albumin-mediated uptake effect with OAT1/3, six highly protein-bound OAT1/3 substrates (fraction unbound in plasma (*f*_u,p_) < 0.2) were shortlisted from a previously published database of renally secreted and excreted OAT1/3 compounds.^42^ Three highly protein-bound compounds were dual substrates of OAT1/3 (olmesartan, bumetanide, and 4-pyridoxic acid) and three were OAT3-specific (rivaroxaban, furosemide, and rosuvastatin). In addition, two weakly protein-bound (*f*_u,p_ > 0.9) specific substrates for OAT1 (adefovir) and OAT3 (oseltamivir carboxylate) were included as potential negative controls for the study of the albumin-mediated uptake effect on OAT1/3 transporters.

Several experimental conditions were investigated: (A) 0% HSA (control), (B) 1% HSA, (C) 4% HSA, and (D) 0% HSA plus probenecid 300 μM (strong clinical inhibitor of OAT1/3^43^). In the presence of 1 and 4% HSA, the unbound substrate concentration was kept constant to that of the control phase based on preliminary experiments (details are given in the Supporting Information) by measuring the fraction unbound of the substrate in the incubation medium (*f*_u,inc_) prior to the experiments. Keeping the unbound concentration constant prevented any plausible difference in the extent of transporter saturation between control and HSA conditions while improving the analytical sensitivity of the assay. The unbound substrate concentration used was kept below the reported Michaelis–Menten constant listed in Table S1. It was assumed that no protein binding occurs in the protein-free control and probenecid conditions (*f*_u,inc_ = 1). Three independent experiments performed in triplicates each were conducted for all conditions and substrates.

Prior to the start of the transporter uptake assay, the confluency of the ciPTEC monolayer on the 24-well plate was verified visually using a microscope. If >80% confluent, the assay proceeded by first removing the culture media and rinsing the cell monolayer twice with 800 μL of HBSS supplemented with 10 mM HEPES (uptake buffer) at pH 7.4 and 37 °C. Thereafter, the cell monolayer was preincubated with uptake buffer for 15 min at 37 °C and removed prior to the start of the experiment. The transporter uptake study was initiated by incubating 300 μL of substrate-containing uptake buffer (including HSA or probenecid depending on the experimental condition investigated) on the ciPTEC monolayer at 37 °C while being shaken at 300 rpm. After various designated time points between 0.5 and 5.0 min (within the linear uptake range), the uptake was terminated by removing the substrate-containing uptake buffer and rinsing the cells with ice-cold uptake buffer thrice. Cells were lysed with distilled water and stored at −80 °C overnight. To prepare the sample for analysis, aliquots of the cell lysate were quenched with acetonitrile containing the internal standard gemfibrozil (0.5 μM) or buspirone (0.05 μM). Samples were vortexed and centrifuged at 16,000 rpm for 30 min to remove any protein before quantifying the intracellular substrate concentration by liquid chromatography and tandem mass spectrometry (LC-MS/MS). Total protein concentration in the cell lysate was measured using the Pierce BCA Protein Assay Kit (ThermoFisher Scientific, Loughborough, U.K.).

### Extent of Albumin Binding

The *f*_u,inc_ for all of the OAT1/3 substrates was measured using the high throughput equilibrium dialysis in the presence of 1 and 4% HSA. Briefly, cellulose membranes with MWCO of 12–14 kDa were submerged in distilled water for 1 h, followed by 20% ethanol/distilled water for at least 20 min. Prior to the start of the experiment, cellulose membranes were rinsed in distilled water and submerged in blank DPBS for 15 min. The HTDialysis device was assembled according to the manufacturer’s instructions (https://www.htdialysis.com). Blank DPBS was first added to the receiver side of the dialysis membranes, followed by DPBS spiked with HSA and the OAT1/3 substrate to the donor side. Each HSA concentration was tested in triplicates. The HTDialysis device was sealed with an adhesive sealing film and incubated in a water bath at 37 °C for 6 h shaken at 150 rpm. Once the assay was completed, aliquots from both receiver and donor wells were transferred to a 96-well plate and stored at −80 °C until analysis. DPBS spiked with the OAT1/3 substrate from both before and after incubation was stored to assess the recovery and stability of the samples. To prepare the sample for analysis, donor and receiver samples were matrix-matched and quenched with ice-cold acetonitrile containing an internal standard (0.5 μM gemfibrozil or 0.05 μM buspirone). Samples were vortexed and centrifuged at 16,000 rpm for 30 min before LC-MS/MS analysis.

### LC-MS/MS Bioanalysis

Substrate quantification was performed using a Waters 2795 high-performance LC (HPLC) system (Waters, Wilmslow, U.K.) coupled to a Quattro Ultima MS (Waters, Wilmslow, U.K.) or an Agilent 1100 HPLC (Agilent Technologies, Stockport, U.K.) coupled to a QTRAP 6500 MS (SCIEX, Macclesfield, U.K.). LC separation was performed using a Luna C18 column (3 μm, 50 × 4.6 mm, Phenomenex, Macclesfield, U.K.) and mobile phases consisting of methanol/water containing 0.5% formic acid or 1.0 mM ammonium acetate at a predetermined gradient (flow rate = 1.0 mL/min, split to deliver 0.25 mL/min to the MS). For MS sample analysis, the electrospray ionization multiple reaction monitoring was operated in negative or positive mode depending on the substrate. A detailed summary of the LC-MS/MS parameters is given in Table S2. Data collection and quantification were performed using QuanLynx software (MassLynx version 4.1, Waters) and Analyst software (version 1.6.3, SCIEX). Calibration curves were prepared using untreated ciPTEC cell lysate spiked with the appropriate drug calibration standards.

### Determination of OAT1/3 Intrinsic Clearance and the Extent of the Albumin-Mediated Uptake Effect

Intracellular uptake (pmol/mg) at every time point was first obtained by normalizing the measured intracellular concentration (pmol/L) against the concentration of the ciPTEC lysate protein per well (mg/L). The CL_int,u_ (μL/min/mg) was then estimated by dividing the gradient of the intracellular uptake versus time profile with the unbound substrate concentration (μM). Only time points during the linear uptake phase were considered. The passive diffusion clearance (CL_int,u,passive_) for each substrate was based on measured CL_int,u_ in the presence of 300 μM probenecid (HSA-free). Finally, the active OAT1 or OAT3-mediated CL_int,u_ (CL_int,u,active_) was calculated by subtracting the CL_int,u,passive_ from the measured CL_int,u_ in the control or HSA phase. The extent of the albumin-mediated uptake effect for every OAT1/3 substrate was estimated by calculating the ratio (*R*) of OAT1/3 CL_int,u,active_ in the presence and absence of HSA.
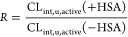
1Where CL_int,u,active_ (+HSA) and CL_int,u,active_ (−HSA) represent the CL_int,u,active_ of an OAT1/3 substrate measured in the presence and absence of HSA, respectively. To understand the relationship between the albumin-mediated uptake effect on CL_int,u,active_ (*R* value) and the extent of protein binding at 1 and 4% HSA, linear regression analysis was performed on log_10_-transformed *R* value versus log_10_-transformed *f*_u,inc_ for the OAT1 and OAT3 data sets. The extra-sum-of-squares *F*-test was used to assess for a statistically significant slope and for any significant difference between the slope and *Y*-intercept of OAT1 and OAT3 data sets. A *p*-value <0.05 was considered statistically significant. All linear regression and data analyses were performed using Graphpad Prism 10.1.1 (Graphpad Software, La Jolla, CA) and Microsoft Excel (Microsoft, Redmond, WA).

### Facilitated-Dissociation Model

A facilitated-dissociation model was adapted^3,15,22^ and applied to experimental data generated here. The increase in CL_int,u_ due to the inclusion of HSA was defined as shown in eq 2:
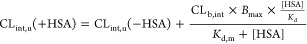
2where *K*_d,m_ (μM) and *B*_max_ (μM) represent the dissociation constant and binding capacity of albumin with the surface of the cell membrane, respectively. CL_b,int_ represents the intrinsic uptake clearance of the additional unbound ligand, which dissociates from the ligand–albumin complex. *K*_d_ represents the dissociation constant of the ligand (OAT1/3 substrate in this scenario) from albumin and was estimated using eq 3 and nonlinear regression of experimental measurements of *f*_u,inc_.
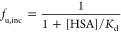
3As CL_b,int_ and *B*_max_ were individually indistinguishable, eq 4 included the product of both parameters, referred to as *V*_b,max_.
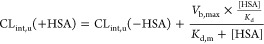
4Where *V*_b,max_ (pmol/min/mg) represents the clearance capacity for a ligand that is transported via the facilitated-dissociation mechanism after dissociating from the albumin–ligand complex. In eqs 2 and 4, *K*_d,m_ is a ligand-independent constant that differs depending on the cell type, whereas *K*_d_ and *V*_b,max_ are ligand-dependent parameters. *V*_b,max_ and *K*_d,m_ were simultaneously estimated with eq 4 using nonlinear least-squares regression fitting on Graphpad Prism. Due to a smaller number of OAT1 (versus OAT3) substrates being investigated, we were unable to estimate *K*_d,m_ for ciPTEC-OAT1. Therefore, the initial estimation of *V*_b,max_ and *K*_d,m_ (ligand-independent constant) was performed using solely data for the OAT3 substrates. Subsequently, *K*_d,m_ for ciPTEC-OAT1 was assumed to be the same as that for ciPTEC-OAT3, and *V*_b,max_ was then estimated for the OAT1 substrates. This is a reasonable assumption as the binding affinity and capacity of HSA to the membrane surface is likely to be similar between the ciPTEC-OAT1 and ciPTEC-OAT3.

Alternatively, eq 2 was expressed as a ratio of OAT1/3 CL_int,u_ in the presence and absence of HSA (*R*) to reflect the extent of the albumin-mediated effect on CL_int,u_, as shown in eq 5:
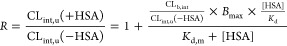
5

By assuming that the ratio of CL_b,int_ to CL_int,u_(−HSA) and *B*_max_ are ligand-independent constants, eq 5 may be further reduced to
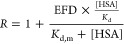
6EFD is a ligand-independent constant representing the extent of facilitated dissociation  for each transporter.^22^ Similar to eq 2, EFD and *K*_d,m_ were first estimated using the fold change in CL_int,u_ for the OAT3 substrates in the presence of 1 and 4% HSA (*R* value). Afterward, *K*_d,m_ of ciPTEC-OAT1 was assumed equal to ciPTEC-OAT3 in order to estimate the EFD for OAT1.

### Bottom-Up Prediction of Renal Clearance

The relative expression and relative activity factor (REF and RAF) are unitless scalers that account for *in vivo* versus *in vitro* differences in the abundance and activity of OAT1/3 and are used for IVIVE of *in vitro* CL_int,u,active_. REF was derived from the OAT1/3 expression ratio between the *in vivo* human kidney cortex to the *in vitro* ciPTEC-OAT1/3, as shown in eq 7:
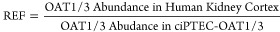
7Absolute abundance of OAT1 and OAT3 in the human kidney cortex was previously quantified by our group using the filter-aided sample preparation protocol and global proteomic analysis.^44^ A similar approach was applied here to quantify the abundance of the ciPTEC-OAT1 and ciPTEC-OAT3 (details are given in the Supporting Information).

RAF is the ratio of the CL_int,u,active_ of a respective OAT1 or OAT3-specific reference compound derived from *in vivo* clinical data to that measured with *in vitro* ciPTEC-OAT1/3:
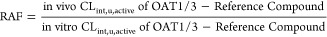
8Adefovir and oseltamivir carboxylate are the FDA-recommended clinical probe substrates of OAT1 and OAT3, respectively,^43^ and were therefore selected as the OAT1/3-specific reference compounds in this study. The extrapolation of *in vitro* CL_int,u,active_ to the *in vivo* CL_int,sec_ using the REF and RAF approach is shown in eqs 9 and 10, respectively.
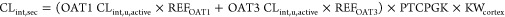
9
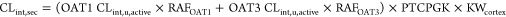
10Where PTCPGK and KW_cortex_ represent the number of proximal tubular cells per gram of kidney cortex (99.4 million PTC/g kidney^45^) and the weight of both kidney cortexes (169 g^39^), respectively. Units of CL_int,u,active_ were expressed as μL/min/10^6^ cells after normalizing by the total protein content of ciPTEC (0.30 and 0.32 mg/10^6^ cells for ciPTEC-OAT1 and ciPTEC-OAT3, respectively). A value of 60 million PTCPGK is commonly used,^32,39,46,47^ but the observed value as high as 209 million PTCPGK has been reported.^37^ In this study, a value of 99.4 million PTCPGK was applied based on the most recent meta-analysis.^45^ A local sensitivity analysis using 60 or 209 million PTCPGK was performed to assess the impact on predicted CL_r_. Finally, plasma secretion clearance (CL_sec_) and plasma renal clearance (CL_r_) were predicted using eqs 11 and 12, respectively.
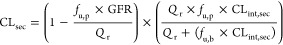
11
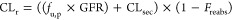
12Where GFR is the glomerular filtration rate of a healthy adult (120 mL/min), Q_r_ is the renal blood flow (1008 mL/min), *f*_u,b_ is the fraction unbound in blood, and *F*_reabs_ is the fraction of drug reabsorbed from the kidney tubules back into the systemic circulation. *F*_reabs_ was assumed to be zero, considering that all of the OAT1/3 substrates investigated were significantly renally secreted (ratio of CL_r_ to *f*_u,p_ × GFR > 1.5). The predicted contribution of renal secretion by the OAT1 or the OAT3 to the total CL_r_ was calculated as the fraction of predicted OAT1/3 CL_sec_ to the predicted CL_r_. Successful predictions of CL_int,sec_, CL_sec_, and CL_r_ were assessed by applying 2- and 3-fold error criteria on the predicted versus observed fold error. Precision and bias of the predictions were assessed using the root-mean-square error (RMSE) and geometric mean fold error (GMFE), calculated using eqs 13 and 14, respectively.
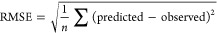
13
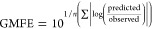
14

## Results

### Extent of Protein Binding to Human Serum Albumin

The fraction unbound in the incubation media (*f*_u,inc_) of the eight OAT1/3 substrates was measured in the presence of 1 and 4% HSA and used to estimate the *K*_d_ (Table 1). The *f*_u,inc_ at 4% HSA (*f*_u,inc-4%_) for all of the substrates in the data set correlated well to the observed fraction unbound in plasma (*f*_u,p_) (majority within 1.5-fold of the observed *f*_u,p_, Figure S1). Five substrates had *f*_u,inc-4%_ < 0.1 and six substrates in the data set had *f*_u,inc-4%_ < 0.2, among which olmesartan was the most highly protein-bound substrate with *f*_u,inc-4%_ of 0.002. Adefovir and oseltamivir carboxylate did not bind appreciably to HSA (*f*_u,inc-4%_ > 0.95) and were, therefore, used as negative controls for investigating the albumin-mediated uptake effect. Recovery and stability of all of the OAT1/3 substrates were greater than 80%.

**Table 1:** Summary of the Measured Human Serum Albumin-Binding Parameters for Eight Selected OAT1/3 Substrates^a^

substrate	*f*_u,inc_ (1%)	*f*_u,inc_ (4%)	*K*_d_ (μM)^b^
olmesartan	0.011 ± 0.001	0.0020 ± 0.0002	1.43 ± 0.10
bumetanide	0.141 ± 0.012	0.029 ± 0.003	21.1 ± 1.4
furosemide	0.162 ± 0.030	0.038 ± 0.002	26.1 ± 1.6
rivaroxaban	0.217 ± 0.006	0.096 ± 0.007	55.0 ± 4.3
4-pyridoxic acid	0.298 ± 0.005	0.079 ± 0.006	56.4 ± 2.5
rosuvastatin	0.435 ± 0.041	0.180 ± 0.009	126.7 ± 5.6
oseltamivir carboxylate	0.973 ± 0.039	0.983 ± 0.018	26300 ± 18900
adefovir	0.999 ± 0.019	0.977 ± 0.062	27100 ± 35200

a Data presented as mean ± standard deviation (SD).

b Dissociation constant of the ligand (OAT1/3 substrate) from albumin (*K*_d_) was estimated using eq 3.

### Effect of Albumin on Renal OAT1/3 Activity

**Figure 1:**
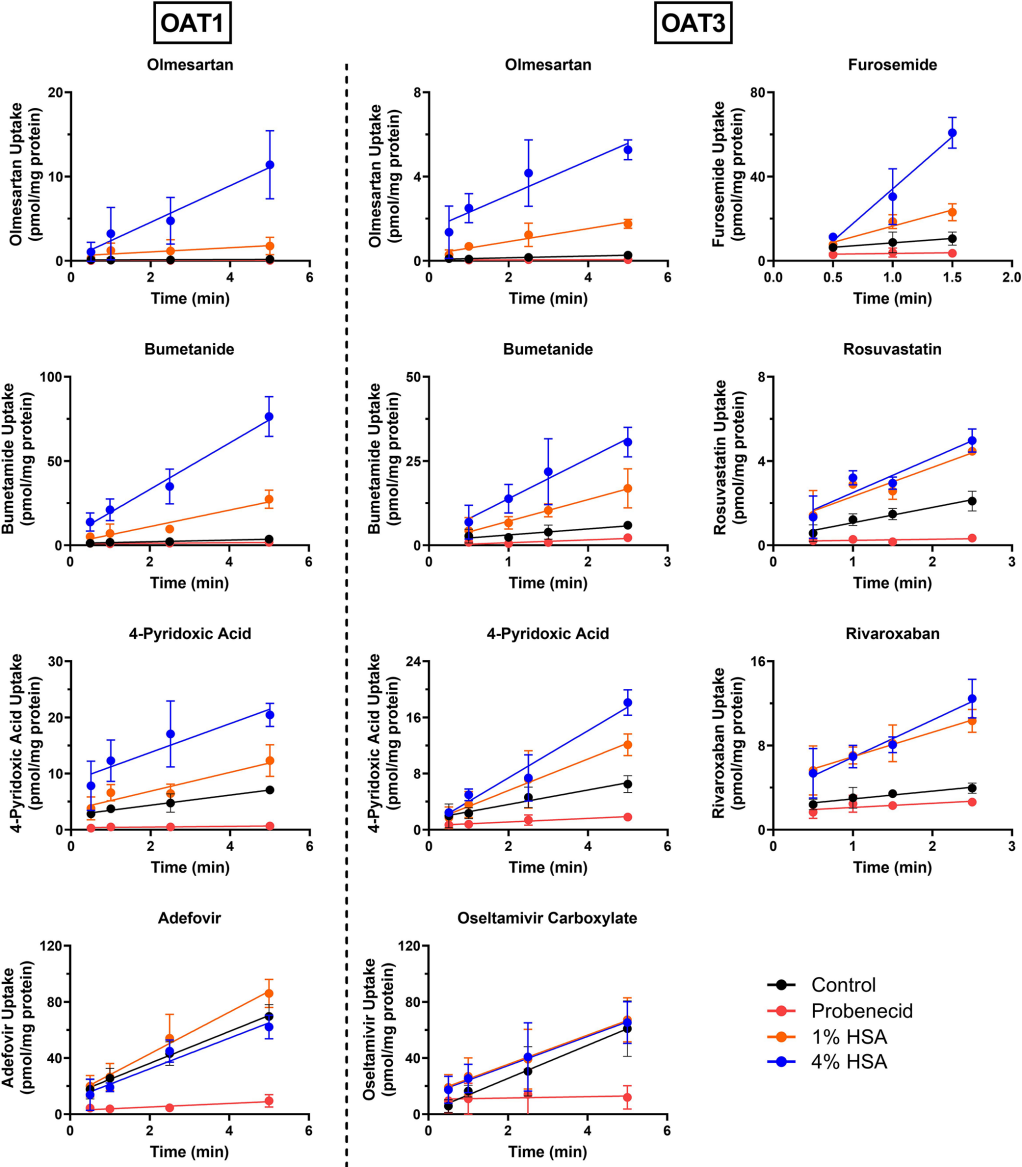
Effect of human serum albumin (HSA) on the total intracellular uptake vs time profile of eight OAT1/3 substrates. Each colored symbol represents the intracellular uptake measured during the control (0% HSA): black, probenecid 300 μM: red, 1% HSA: orange, and 4% HSA conditions: blue (mean ± SD of three independent replicates). As the unbound concentration of the OAT1/3 substrate was kept constant between conditions, any increase in the slope of the best-fit linear regression line in the presence of 1 and 4% HSA represents an equivalent fold increase in the unbound intrinsic clearance as a result of the albumin-mediated uptake effect.

Using the information on the extent of binding in a particular HSA condition (measured *f*_u,inc_), the unbound substrate concentration incubated with the ciPTEC-OAT1/3 was kept constant in the control, 1% HSA, and 4% HSA conditions. Thus, changes in the slope of the uptake versus time profiles shown in Figure 1 reflect a shift in CL_int,u_ across various albumin conditions. The inclusion of probenecid significantly reduced the level of the OAT1/3-mediated uptake of the substrates in ciPTEC-OAT1/3, verifying substrate specificity for OAT1 or OAT3 and allowing the estimation of passive diffusion (CL_int,u,passive_). Olmesartan, bumetanide, and 4-pyridoxic acid were confirmed to be dual OAT1/3 substrates, consistent with the published literature. Furosemide, rivaroxaban, rosuvastatin, and oseltamivir carboxylate did not show any appreciable uptake in ciPTEC-OAT1, and adefovir was not an OAT3 substrate (Figure S2). For these compounds, the inclusion of probenecid in these conditions did not lead to any significant change in the uptake from the control, in agreement with previously published data. Due to conflicting evidence in the literature for the transport of furosemide by OAT1, additional experiments investigating a range of furosemide concentrations were performed and demonstrated the absence of OAT1 uptake (Figure S3).

**Figure 2:**
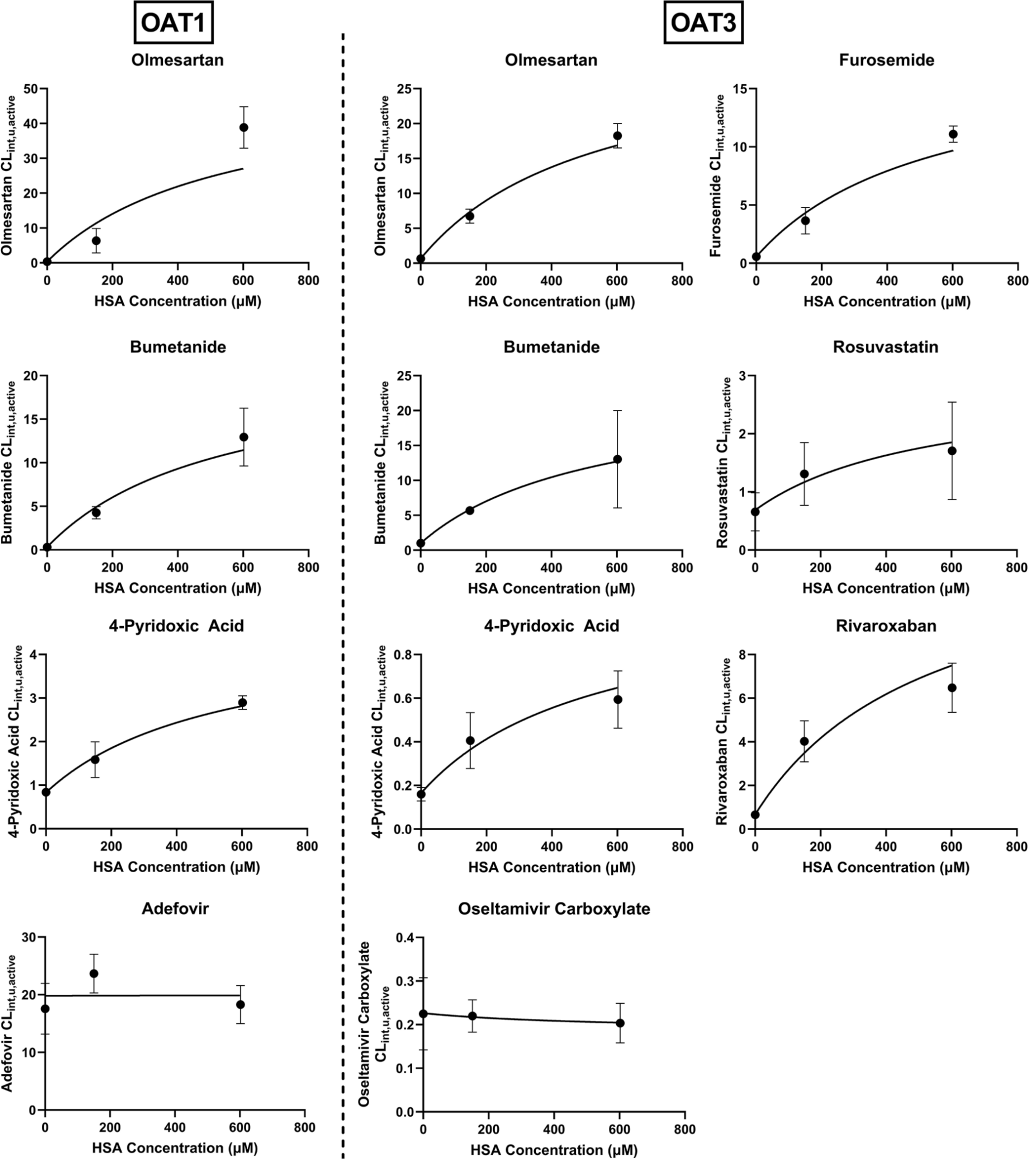
OAT1 and OAT3 unbound intrinsic clearance (CL_int,u,active_) of eight OAT1/3 substrates measured in the presence and absence of 1 and 4% HSA. The symbols represent the measured CL_int,u,active_ (mean ± SD of three independent replicates), and solid fitted lines represent the fit for the facilitated-dissociation model (eq 4). Adefovir and oseltamivir carboxylate are weakly protein-bound substrates and showed no effect of albumin on their uptake. When eq 4 was fitted to the OAT1 data set, *V*_b,max_ was estimated with a fixed *K*_d,m_ value estimated from the OAT3 data set.

With the inclusion of 1 and 4% HSA, highly protein-bound substrates showed an increase in the measured CL_int,u_, providing evidence of an albumin-mediated uptake effect with both OAT1 and OAT3 (Figure 1). In contrast, the weakly protein-bound negative controls (adefovir and oseltamivir carboxylate) did not show any significant change in the uptake with the inclusion of HSA. The presence of a higher concentration of HSA in the incubation medium resulted in a greater increase in the measured CL_int,u,active_ (Figure 2); these trends were consistent among all of the highly protein-bound substrates. Fitting of the facilitated-dissociation model to the experimental CL_int,u,active_ and *K*_d_ data (eq 4) estimated the dissociation constant between albumin and the surface of ciPTEC (*K*_d,m_ = 542 ± 217 μM, Table S3). There was a minimal difference in the estimate of *K*_d,m_ and *V*_b,max_ when using CL_int,u_ (OAT1/3 active uptake plus passive diffusion) or CL_int,u,active_ (OAT1/3 active uptake only) (Table S3); hence, the latter was used for consistency with analyzing the extent of the albumin-mediated uptake effect on the active OAT1/3.

**Figure 3:**
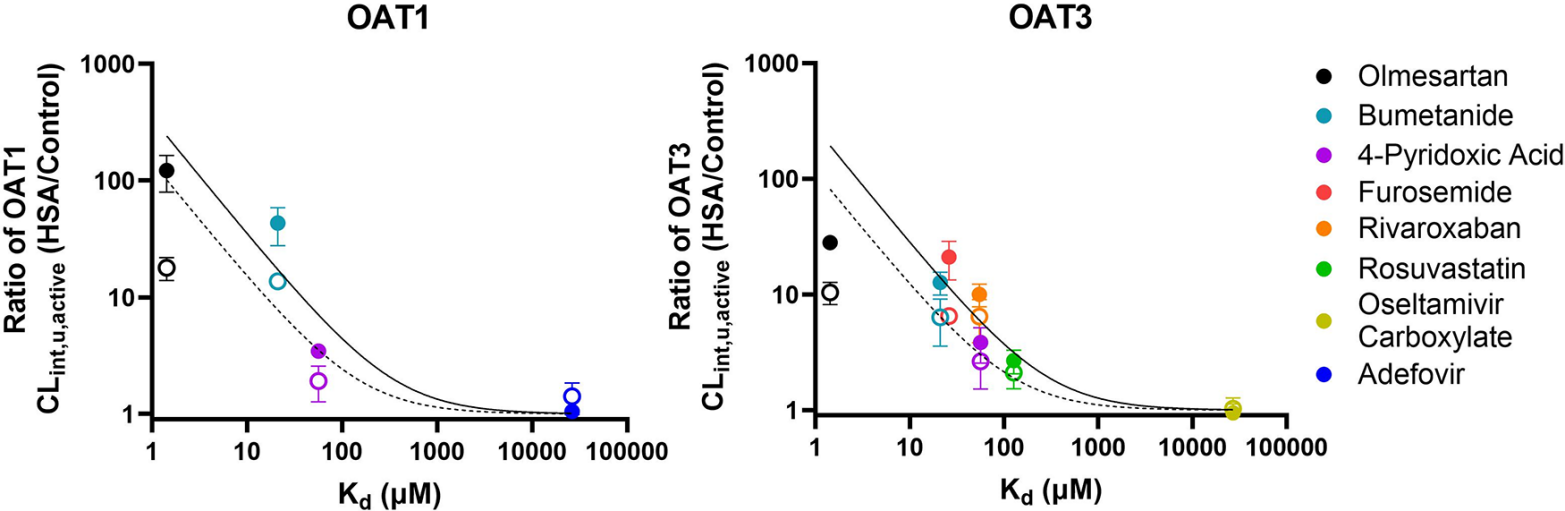
Correlation between the ratio of OAT1/3 unbound intrinsic clearance (CL_int,u,active_(+HSA)/CL_int,u,active_(−HSA)) and the albumin–ligand dissociation constant (*K*_d_). The empty and solid symbols represent the ratio of CL_int,u,active_ measured with 1 and 4% HSA versus the control phase, respectively (mean ± SD of three independent replicates). Each colored symbol represents a different OAT1/3 substrate: olmesartan: black, bumetanide: teal, 4-pyridoxic acid: purple, furosemide: red, rivaroxaban: orange, rosuvastatin: green, oseltamivir carboxylate: yellow, and adefovir: blue. Dashed and solid lines represent the fitted line obtained using the facilitated-dissociation model (eq 6) for the 1 and 4% HSA conditions, respectively. When eq 6 was fitted to the OAT1 data set, a fixed *K*_d,m_ value was used based on the OAT3 analysis.

In general, for dual OAT1/3 substrates, a greater albumin-mediated uptake effect on CL_int,u,active_ was noted for ciPTEC-OAT1 than for ciPTEC-OAT3 cells, demonstrated by a larger fold-increase relative to that of no albumin control (Table 2). Additionally, there was an inverse correlation between the extent of the albumin-mediated uptake effect and the extent of drug-albumin binding (Figure 3). Substrates with a smaller *K*_d_ had a greater albumin-mediated effect on their uptake and a larger fold increase in CL_int,u,active_ (*R* value) (Figure 3). For example, olmesartan, the most highly bound OAT1/3 substrate in our data set (*f*_u,inc-4%_ = 0.002), had a 122- and 28-fold increase in the OAT1 and OAT3 CL_int,u,active_, respectively, in 4% HSA (Table 2). Bumetanide (*f*_u,inc-4%_ = 0.029) was the drug with the second largest effect, with a 43.2- and 12.8-fold increase in OAT1 and OAT3 CL_int,u,active_, respectively. Among the six highly protein-bound substrates investigated, rosuvastatin had the largest *f*_u,inc-4%_ of 0.18 and the smallest increase in OAT3 CL_int,u,active_ of 2.7-fold. Simultaneous fitting of the reduced facilitated-dissociation model (eq 6) to fold increase in OAT3 CL_int,u,active_ and K_d_ estimated a *K*_d,m_ of 520 ± 434 μM and the extent of facilitated dissociation (EFD) of 513 ± 265 μM (Table S4). This estimate of *K*_d,m_ was comparable to the value obtained using the full facilitated-dissociation model (eq 4, Table S3). The estimated OAT1 EFD of 641 ± 137 μM was larger than OAT3, in agreement with the greater albumin-mediated uptake effect seen with OAT1 than OAT3 for dual OAT1/3 substrates.

**Table 2:** Summary of *In Vitro* OAT1/3 Unbound Intrinsic Clearance (CL_int,u,active_) in the Presence of 1 and 4% Human Serum Albumin (HSA)^a^

	CL_int,u,active_ (μL/min/mg)			ratio of CL_int,u,active_ (*R*) (HSA/control)	
substrates	control	1%	4%	1%	4%
OAT1					
olmesartan	0.34 ± 0.13	6.45 ± 3.52	38.9 ± 6.0	17.9 ± 4.0	122 ± 42
bumetanide	0.32 ± 0.08	4.26 ± 0.70	12.9 ± 3.31	13.7 ± 1.4	43.2 ± 15.5
4-pyridoxic acid	0.84 ± 0.07	1.59 ± 0.41	2.90 ± 0.16	1.92 ± 0.65	3.46 ± 0.47
adefovir	17.6 ± 4.4	23.7 ± 3.4	18.3 ± 3.3	1.41 ± 0.43	1.05 ± 0.07
OAT3					
olmesartan	0.65 ± 0.04	6.74 ± 1.00	18.3 ± 1.8	10.5 ± 2.3	28.1 ± 3.0
bumetanide	1.00 ± 0.40	5.68 ± 0.54	13.0 ± 6.9	6.4 ± 2.8	12.8 ± 2.9
4-pyridoxic acid	0.16 ± 0.03	0.41 ± 0.13	0.59 ± 0.13	2.64 ± 1.12	3.85 ± 1.30
furosemide	0.56 ± 0.16	3.65 ± 1.15	11.1 ± 0.7	6.52 ± 0.90	21.1 ± 7.7
rivaroxaban	0.65 ± 0.12	4.03 ± 0.95	6.47 ± 1.13	6.44 ± 2.61	10.0 ± 2.2
rosuvastatin	0.66 ± 0.33	1.31 ± 0.54	1.71 ± 0.84	2.12 ± 0.58	2.68 ± 0.62
oseltamivir carboxylate	0.23 ± 0.08	0.22 ± 0.04	0.20 ± 0.05	1.04 ± 0.24	0.95 ± 0.18

a Data presented as mean ± SD of three independent replicates.

**Figure 4:**
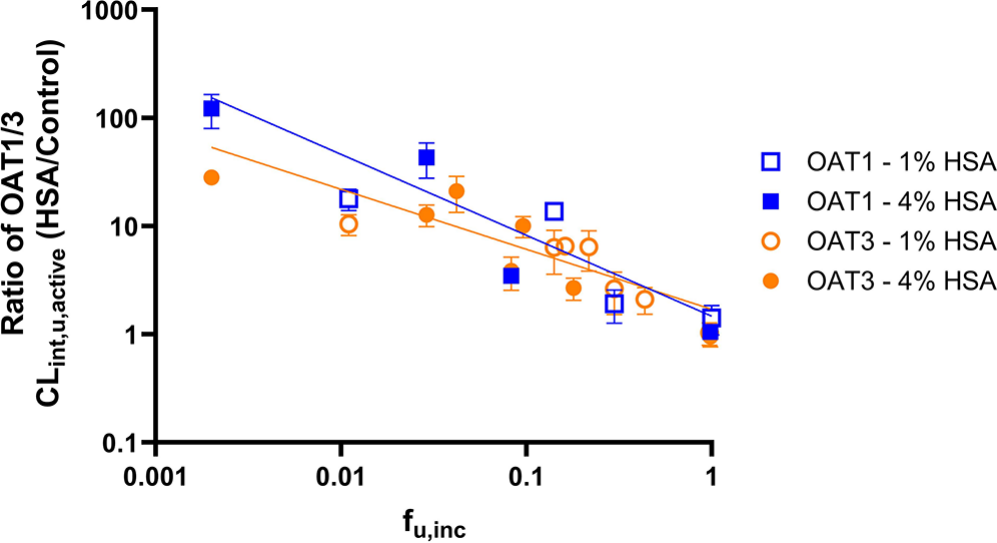
Correlation between the ratio of OAT1/3 unbound intrinsic clearance (*R* = CL_int,u,active_(+HSA)/CL_int,u,active_(−HSA)) and the fraction unbound in the incubation system (*f*_u,inc_) measured with 1 and 4% HSA. Linear regression analysis was performed on the log_10_-transformed *R* value and log_10_-transformed *f*_u,inc_ data with a statistically significant difference between OAT1 and OAT3 data sets (*F*_(2,62)_ = 3.89, *p*-value = 0.026). The best-fit values for the OAT1 and OAT3 data sets are log_10_(*Y*_OAT1_) = −0.73(log_10_*X*) + 0.087 (RMSE = 0.29, *r*^2^ = 0.84) and log_10_(*Y*_OAT3_) = −0.54(log_10_*X*) + 0.18 (RMSE = 0.23, *r*^2^ = 0.74) The empty and solid symbols represent the ratio of CL_int,u,active_ measured with 1 and 4% HSA versus the control phase, respectively (mean ± SD of three independent replicates). OAT1 and OAT3 data sets are represented by the blue and orange symbols/lines, respectively.

Further linear regression analysis revealed a statistically significant slope (*p*-value <0.0001) in the relationship between the log_10_-transformed fold-increase in OAT1 and OAT3 CL_int,u_,_active_ (*R*) and the fraction unbound of substrates in the presence of 1 and 4% HSA (*f*_u,inc_). Best-fit values of the estimated slope and *Y*-intercept for the OAT1 and the OAT3 data sets were statistically significantly different (*F*_(2,62)_ = 3.89, *p*-value = 0.026), confirming the difference in the extent of the albumin-mediated uptake effect observed for the OAT1 and the OAT3 (Figure 4). Two equations were derived for the OAT1 and the OAT3 data sets where log_10_(*R*_OAT1_) = −0.73log_10_(*f*_u,inc_) + 0.087 (RMSE = 0.29, *r*^2^ = 0.84) and log_10_(*R*_OAT3_) = −0.54log_10_(*f*_u,inc_) + 0.18 (RMSE = 0.23, *r*^2^ = 0.74). The relatively strong correlation (*r*^2^ > 0.7) between OAT1/3 R values versus *f*_u,inc_ and the low RMSE (<1) suggest that the extent of the albumin-mediated uptake effect of OAT1/3 substrates may be estimated using each equation for the corresponding transporter and measured *f*_u,inc_.

### Improvement to the Bottom-Up Prediction of OAT1/3-Mediated Secretion and Renal Clearance with the Inclusion of Data Obtained in the Presence of Albumin

The REF values for the two transporters, OAT1 and OAT3, were derived by quantifying the absolute abundance of the transporters in ciPTEC-OAT1/3 using global proteomics. The expression of OAT1/3 in the ciPTEC cell lines was much lower than the values reported in the *in vivo* kidney cortex^44^ (Table 3), resulting in REF for OAT1 and OAT3 of 8.0 and 32.0, respectively. The CL_int,u,active_ of adefovir (OAT1-specific probe) and oseltamivir carboxylate (OAT3-specific probe) measured in the ciPTEC (in the absence of albumin) underestimated the *in vivo* CL_int,u,active_ value back-calculated from clinical measurements of CL_r_, resulting in RAF values of 2.0 and 161.2 for OAT1 and OAT3, respectively.

**Table 3:** Derivation of the Relative Expression Factor (REF) and the Relative Activity Factor (RAF) for OAT1 and OAT3

	transporter protein abundance (pmol/mg protein)			OAT1/3 unbound intrinsic clearance (μL/min/mg)		
	human kidney cortex^a^	ciPTEC-OAT1/3^b^	REF^c^	*In vivo*^d^	*In vitro*^e^	RAF^c^
OAT1	10.2 ± 10.1	1.28 ± 0.32	8.0	35.2	17.6 ± 4.4	2.0
OAT3	9.6 ± 9.8	0.30 ± 0.14	32.0	36.2	0.23 ± 0.08	161.2

a Mean ± SD abundance of 15 (OAT1) and 16 (OAT3) kidney cortex samples.^44^

b Mean ± SD of four replicates.

c REF and RAF calculated using eqs 7 and 8, respectively.

d Average unbound intrinsic clearance back-calculated from published renal clearance of adefovir^48−50^ and oseltamivir carboxylate.^51^

e Mean ± SD of three independent replicates.

**Figure 5:**
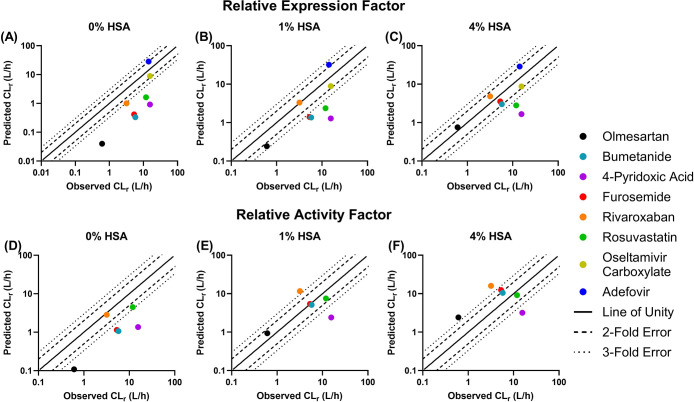
Comparison of predicted versus observed plasma renal clearance (CL_r_) obtained using the relative expression factor (top panels) or the relative activity factor approach (bottom panels). Predictions are based on measurements of the unbound intrinsic clearance of OAT1 and/or OAT3 in the control (A and D) and in the presence of 1% (B and E) and 4% human serum albumin (C and F). Each colored symbol represents a different OAT1/3 substrate: olmesartan: black, bumetanide: teal, 4-pyridoxic acid: purple, furosemide: red, rivaroxaban: orange, rosuvastatin: green, oseltamivir carboxylate: yellow, and adefovir: blue. Solid, dashed, and dotted lines represent the line of unity, 2-fold error criterion, and 3-fold error criterion, respectively.

**Figure 6:**
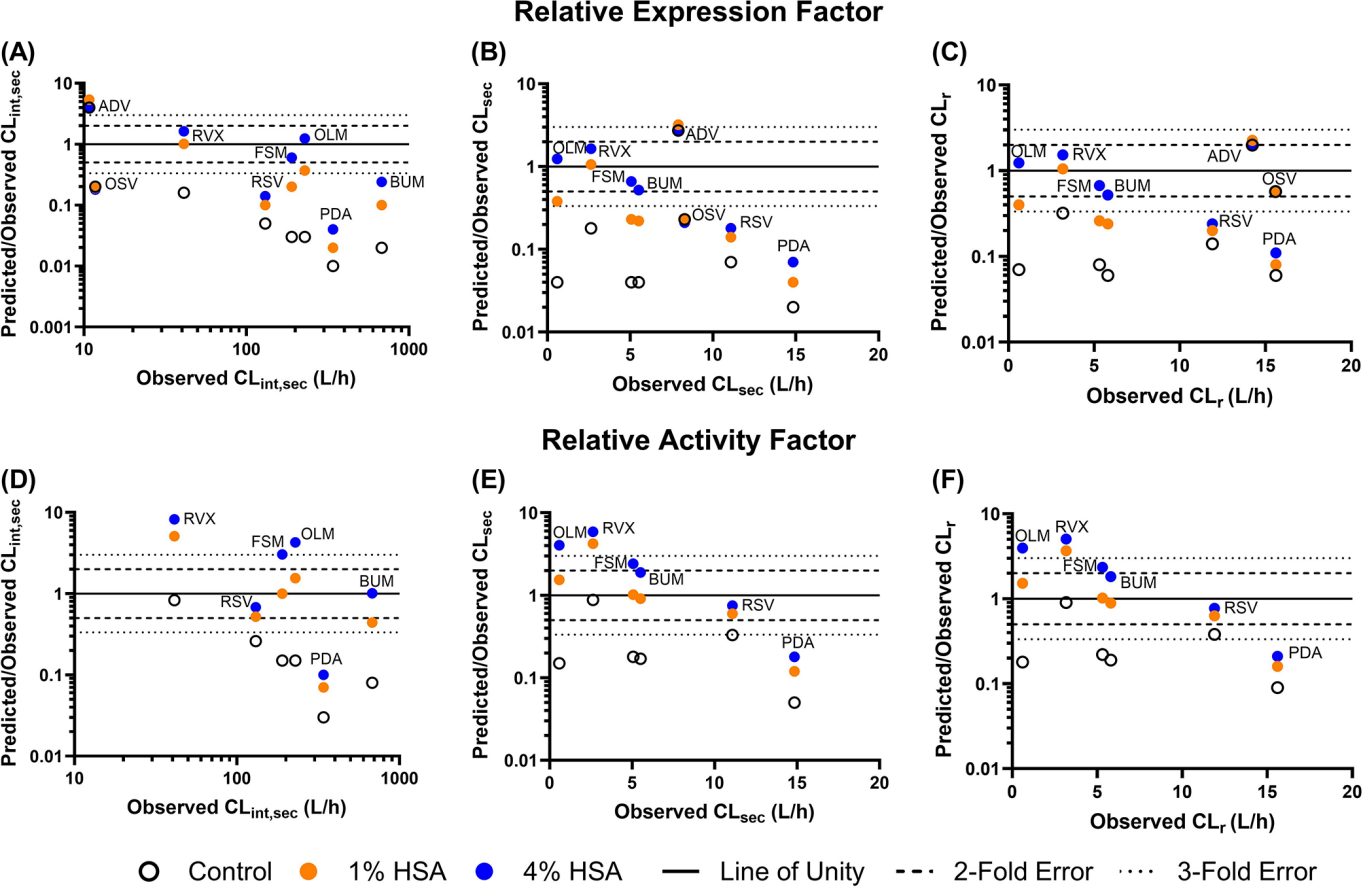
Fold difference in predicted/observed versus observed *in vivo* intrinsic secretion clearance (CL_int,sec_, A and D), plasma secretion clearance (CL_sec_, B and E), and plasma renal clearance (CL_r_, C and F), obtained using the relative expression factor (top panels) or the relative activity factor approach (bottom panels). The colored symbols represent the 0% (black), 1% (orange), and 4% human serum albumin (blue) conditions where each set of colored symbols represents an OAT1/3 substrate as abbreviated by ADV, adefovir; BUM, bumetanide; FSM, furosemide; OLM, olmesartan; OSV, oseltamivir carboxylate; PDA, 4-pyridoxic acid; RSV, rosuvastatin; and RVX, rivaroxaban.

Using the REF and RAF approaches, the *in vivo* intrinsic secretory clearance (CL_int,sec_), secretion, and renal clearance (CL_sec_ and CL_r_) were predicted from transporter kinetic measurements in the presence and absence of HSA (eqs 9 to 12, values listed in Table S5). When the RAF approach was applied, OAT1/3 probe substrates (adefovir and oseltamivir carboxylate) were excluded from the IVIVE analysis, as their clinical data were used to derive the RAF scaling factor. A general underprediction of CL_r_ was noted with both REF and RAF approaches when the control data (no albumin) were used, with a clear improvement in the prediction of CL_r_ when albumin was included during measurements of *in vitro* transporter kinetic parameters (Figure 5). IVIVE of CL_int,u,active_ measured in the absence of HSA underpredicted the *in vivo* CL_int,sec_ by more than 5-fold for all of the highly bound substrates (except rivaroxaban) with both REF and RAF approaches (Table S5). In contrast, the use of data obtained in the presence of HSA improved the bottom-up prediction of CL_int,sec_, CL_sec_, and CL_r_ (Figure 6). The number of OAT1/3 substrates for which CL_r_ was predicted within 2-fold increased with a higher % albumin condition, e.g., 2/8 for 1% HSA compared to 5/8 predicted when 4% HSA data were used with the REF approach (Figure 5, Table S6). Similar improvements upon inclusion of albumin were observed with predicted CL_int,sec_ and CL_sec_, and the RAF approach, although with some tendency for overprediction with 4% HSA (Figure 6). The accuracy of predicted CL_r_ improved with the inclusion of HSA, as GMFE decreased from 6.8 (control) to 3.2 (1% HSA) and 2.3 (4% HSA) with the REF approach and 4.1 (control) to 2.0 (1% HSA) and 2.9 (4% HSA) with the RAF approach. The inclusion of data obtained in the presence of albumin had the greatest impact on the predictive performance of OAT1/3 substrates with a lower observed CL_r_ or CL_sec_ (olmesartan, bumetanide, rivaroxaban and furosemide, Figure 6). CL_r_, CL_sec_, and CL_int,sec_ of 4-pyridoxic acid (OAT1/3 endogenous biomarker, observed CL_r_ of 15.6 L/h) were consistently underpredicted (predicted values <20% of observed) regardless of albumin inclusion and the use of the RAF/REF approach, although slight improvements were seen with the data obtained in the presence of 4% HSA. Rosuvastatin clearance parameters were equally poorly predicted with the REF approach (4- to 7-fold underprediction). Varying the value of PTCPGK from 60 to 209 million PTC/g as a local sensitivity analysis resulted in a minimal change in the bias of predicted CL_r_ at the 4% HSA condition (Table S7).

In general, the inclusion of HSA increased the predicted percentage transported by OAT1 (versus that by OAT3) (Figure S4) due to a larger albumin-mediated uptake effect on OAT1 than that on OAT3. The percentage transported by OAT1 increased by 2- to 4-fold upon the inclusion of 4% HSA versus the control for dual OAT1/3 substrates (bumetanide, olmesartan and 4-pyridoxic acid). The predicted contribution of OAT1 with the REF scaler varied between 19 and 33% for the dual OAT1/3 substrates using measured data in the presence of 4% HSA. In contrast, considerably larger RAF_OAT3_ of 161 versus the REF_OAT3_ of 32.0 resulted in a heavily skewed contribution toward OAT3 (<5% transported by OAT1) with the RAF approach (Figure S4), likely causing the overprediction of CL_int,sec_, CL_sec_, and CL_r_ when using 4% HSA data combined with RAF (Figure 6).

## Discussion

Numerous studies have recently demonstrated the albumin-mediated uptake effect on the activity of hepatic OATP1B1/3 transporters,^3,5^ and reported that the inclusion of albumin or whole plasma in the medium of hepatocyte/hepatic transporter assays improves the IVIVE of hepatic clearance.^6,7,30,52^ Some studies have explained this albumin-mediated phenomenon by the facilitated-dissociation model,^3,6,22^ hypothesizing that the albumin–ligand complex interacts with a receptor on the cell surface, leading to a conformational change in albumin and the dissociation of the ligand (transporter substrate), hence resulting in an additional unbound substrate available for uptake into the cell. However, due to the varying experimental conditions employed by these studies, it is uncertain if the albumin-mediated uptake effect is due to albumin per se (or other components in human plasma) and to what extent the effect depends on the cell type used (hepatocyte or immortalized cell lines). Equally, we wondered whether this phenomenon would also occur with other uptake transporters, including renal OAT1/3. Similar to hepatocytes, renal proximal tubular cells are involved in the transport of albumin. Albumin that has been filtered at the glomerulus is subsequently reabsorbed from the renal tubules back into the bloodstream via the megalin/cubulin receptor complex and neonatal Fc receptor (FcRn).^53,54^ Therefore, it is plausible that the albumin-mediated uptake effect may also occur with renal uptake transporters expressed on the basolateral membrane of proximal tubular cells. In this study, we investigated whether the albumin-mediated uptake effect exists with the renal OAT1 and OAT3 transporters and its implication on the IVIVE of renal secretion and total renal clearance for OAT1/3 substrates.

In the current study, we observed a substantial increase in the intracellular uptake of six highly protein-bound substrates in ciPTEC-OAT1/3 cell lines with the inclusion of HSA (Table 2). In order to directly assess the albumin-mediated effect, the unbound substrate concentration was kept constant between the control and HSA conditions. This design enabled us to directly measure a 3.5- to 122-fold increase in OAT1 CL_int,u,active_ and a 2.68- to 28-fold increase in the OAT3 CL_int,u,active_ across the investigated data set with the inclusion of 4% HSA. This increase is inconsistent with the free-drug hypothesis, where the unbound clearance is not expected to change with the inclusion of albumin/protein as only the free (unbound) drug interacts with the transporter. The magnitude of increase in both OAT1 and OAT3 CL_int,u,active_ was correlated with the concentration of HSA included (Figure 2) and the extent of albumin binding (*K*_d_) or the fraction unbound of a drug (Figures 3 and 4). Similar trends were observed in two studies that have investigated the albumin effect on a range of hepatic OATP1B1/3 substrates in human hepatocytes.^3,6^ Furthermore, the absence of any change observed in the CL_int,u,active_ of two weakly protein-bound substrates (adefovir and oseltamivir carboxylate) demonstrates that this phenomenon was conditional in the binding between albumin and the ligand (OAT1/3 substrate). Changes to the passive diffusion of OATP1B1/3 substrates upon the inclusion of albumin/plasma have been suggested.^5,6,8,55^ In our analysis, a minimal difference was noted when the facilitated dissociation model was fitted to total CL_int,u_ (OAT1/3 active uptake plus passive diffusion) or OAT1/3 CL_int,u,active_ data (OAT1/3 active uptake only) (Table S3), corroborating the assumption of no significant changes in passive diffusion upon inclusion of albumin. Additionally, preliminary experiments with 4-pyridoxic acid indicated a minimal change to its passive diffusion clearance upon inclusion of 1% HSA in the probenecid condition (Figure S6).

Recent studies^8,55^ detected residual albumin after experiments with hepatocytes and OATP1B1-transfected human embryonic kidney cells (HEK293), alluding the albumin-mediated uptake phenomenon for a subset of investigated OATP1B1/3 drugs to nonspecific binding of the albumin–ligand complex to the labware or cell surface. Here, we kept the unbound substrate concentration constant between control and albumin conditions by controlling for any binding to HSA (Table 1) and observed negligible nonspecific binding to plasticware (Table S8). It is unsurprising that albumin is bound to the hepatocyte surface considering the physiological role of the liver in albumin synthesis and degradation,^56^ where receptors such as FcRn^57^ facilitate the movement of albumin into/out of the hepatocyte. If these albumin-binding receptors are involved in the facilitated dissociation process, any disease-related changes in the expression of these receptors may modulate the activity of uptake transporters (in addition to potential disease-related changes in the albumin concentration). Based on the facilitated-dissociation model, the interaction of albumin with the cell surface is a necessary element of the albumin-mediated uptake phenomenon. Fitting of the facilitated-dissociation model (eqs 4 and 6) to our experimental data yielded estimates of the dissociation constant between albumin and the surface of ciPTEC (*K*_d,m_ = 520–542 μM) that approached the physiological concentration of albumin. Previous estimates of *K*_d,m_ for human hepatocytes (45.2 μM^3^) and rat hepatocytes (157 μM^15^) suggest a stronger affinity for albumin to the hepatocyte cell surface than for renal proximal tubular cells. In addition to possible differences in affinity, different albumin-binding cell surface receptors may be expressed in the liver and kidney. Various albumin receptors responsible for albumin homeostasis have been identified thus far, including FcRn, cubilin, megalin, and ubiquitous cell surface glycoproteins gp30 and gp18.^58^ Taken together, our findings collectively demonstrate that an albumin-mediated uptake effect, similar to that observed with the hepatic transporters, does indeed occur for renal OAT1/3.

A larger extent of albumin-mediated uptake effect was found with OAT1 than with OAT3 for the three highly protein-bound dual substrates of OAT1/3 (olmesartan, bumetanide, and 4-pyridoxic acid). In addition to the greater ratio of CL_int,u,active_ (HSA/Control), the estimated extent of facilitated dissociation (EFD) was generally larger for OAT1 than OAT3 for dual OAT1/3 substrates. A similar observation was noted with dual substrates of hepatic OATP1B transporters, where OATP1B1 tended to show a greater albumin-mediated uptake effect than OATP1B3.^5^ While the reason for these differences between OAT1 and OAT3 is uncertain, the reported Michaelis–Menten constants (*K*_m_) for dual OAT1/3 substrates (olmesartan and 4-pyridoxic acid) were smaller for the OAT1 transporter than OAT3 (Table S1), suggesting that affinity for the transporter likely plays a role in this observation. Consequently, the inclusion of albumin led to an increase in the predicted *in vivo* percentage transported via OAT1 versus that via OAT3. For example, the percentage transported via OAT1 of olmesartan increased from 7.9% (control phase) to 33.1% (4% HSA). Therefore, the inclusion of HSA may impact the evaluation of the relative contribution of OAT1 versus OAT3 toward renal secretion of dual OAT1/3 substrates and underscores the importance of investigating the inclusion of HSA in *in vitro* assays for both transporters. Our study did not observe appreciable OAT1-mediated transport across multiple concentrations of furosemide despite this drug being an FDA-recommended clinical probe substrate for both OAT1 and OAT3. OAT1 transport of furosemide was previously observed in a study using HEK293 cells overexpressing OAT1^59^ and s2 cells overexpressing OAT1.^60^ However, a more recent study did not observe any transport of furosemide with HEK293-OAT1^32^ and subsequently proposed furosemide to be an OAT3-specific clinical probe.^61^ The conflicting *in vitro* evidence for furosemide necessitates further investigation to evaluate its appropriateness as an OAT1 clinical probe.

Absolute quantification of the abundance of the OAT1/3 transporters in the ciPTEC using global proteomics and measurement of CL_int,u,active_ for the OAT1- and OAT3-specific clinical probe substrates enabled the derivation of both relative expression and relative activity scaling factor (REF and RAF). The expression and, consequently, the measured activity of OAT1/3 in ciPTEC-OAT1/3 were both considerably lower compared to *in vivo*. Therefore, resulting REF and RAF for OAT1 and OAT3 were substantially >1. In comparison, reported proteomic expression and activity of other OAT1/3-overexpressing cells lines (HEK293-OAT1/3) were greater than the *in vivo* level and had REF/RAF < 1.^32,33^ Nevertheless, application of the REF and RAF scalers in this study enabled accurate predictions of CL_sec_ and CL_r_ (with inclusion of HSA) and emphasized the importance of accounting for the relative differences between the *in vitro* and *in vivo* system when performing bottom-up IVIVE of transporter-mediated clearance.

The inclusion of HSA improved the significant underprediction of CL_int,sec_, CL_sec_, and CL_r_ observed when data in the control phase (no albumin) were used, especially for highly protein-bound substrates (Figure 6). GMFE generally decreased from the control to 4% HSA condition for the REF approach, e.g., from 18.4 to 4.0 (CL_int,sec_), 13.3 to 3.0 (CL_sec_), and 6.8 to 2.3 (CL_r_). The REF approach with the 4% HSA condition had the best overall predictive performance of CL_r_ with 62.5 and 75% of investigated substrates predicted within 2- and 3-fold error, respectively. Interestingly, with the RAF approach, there was a tendency to overpredict CL_r_ when using data obtained in the 4% HSA condition, whereas the 1% HSA data showed a slightly better predictive performance (Figure 6, Table S6). While this observation is based on a limited data set of six highly bound substrates, it may also be due to the use of only one OAT1/3-specific probe substrate to derive the RAF scaler in our study. We obtained an RAF_OAT3_ of 161, which was much greater than the REF_OAT3_ of 32.0. Prospective studies may need to consider an average RAF from two specific probe substrates, as performed previously by Mathialagan et al.^32^ for OAT2 and OAT3. Prior studies^32,33^ with a larger number of OAT1/3 substrates had similar predictive performance of CL_r_ despite the absence of albumin in their experiments. However, the majority of drugs in those data sets were moderately to weakly bound substrates (*f*_u,p_ > 0.2), which are not likely to be affected by the albumin-mediated uptake phenomenon. Underprediction of CL_sec_ and CL_r_ of highly protein-bound bumetanide and rosuvastatin in prior studies^32,33^ was comparable to our data in the absence of albumin. The CL_int,sec_, CL_sec_, and CL_r_ of 4-pyridoxic acid were consistently underpredicted (predicted value less than 20% of the observed) regardless of the RAF/REF approach, and with both 1 and 4% HSA data sets. 4-Pyridoxic acid is an endogenous biomarker formed via the breakdown of vitamin B6 by aldehyde oxidase found predominantly in the liver but also in the kidney.^62,63^ An overestimation of 4-pyridoxic acid CL_r_ in clinical studies due to the synthesis of 4-pyridoxic acid in the kidney is unlikely to be the reason for underprediction due to low extrahepatic expression of aldehyde oxidase.^64^

This study was the first to investigate the albumin-mediated uptake phenomenon on the renal OAT1/3 using a data set of substrates with a wide range of plasma protein-binding properties (*f*_u,p_ = 0.0026–0.98) and with two weakly protein-bound OAT1/3 substrates as negative controls. Even for substrates with *f*_u,p_ < 0.1, a broad range was included (olmesartan *f*_u,p_ = 0.0026 to 4-pyridoxic acid *f*_u,p_ = 0.092). Although current findings are based on a data set of 8 drugs, we recommend the inclusion of 4% HSA in *in vitro* OAT1/3 assays, considering the strong evidence of the albumin-mediated effect on these transporters and considerable improvements to IVIVE of OAT1/3-mediated CL_int,sec_, CL_sec_, and CL_r_. As the inclusion of HSA in the OAT1/3 transporter assays may be analytically challenging and not feasible immediately, we propose an empirical relationship to predict the extent of the albumin-mediated uptake effect based on the *f*_u,inc_. Since *f*_u,inc_ measured in the presence of 4% HSA was within 1.5-fold of the observed *f*_u,p_ for all of the investigated compounds (except bumetanide that fell within 2-fold, Figure S1), measurements of OAT1 or OAT3 CL_int,u,active_ in the absence of HSA could be extrapolated to the 4% HSA condition using routine measurements of *f*_u,p_ and the empirical relationship proposed here (Figure 4). As this relationship has been developed using ciPTEC-OAT1/3, future users should validate this relationship with their *in vitro* systems (e.g., HEK293-OAT1/3) before application.

## Conclusions

In conclusion, this study has confirmed the albumin-mediated uptake phenomenon with substrates of renal OAT1/3 using ciPTEC overexpressing OAT1 and OAT3. Albumin-mediated enhancement of the transporter activity was correlated with the extent of protein binding (*K*_d_ and *f*_u,inc_) and did not occur for weakly protein-bound drugs. The increase in the measured unbound CL_int_ upon inclusion of albumin could be explained by the facilitated-dissociation model, where additional unbound drug is available for uptake transport after dissociating from the albumin–drug complex upon interaction with the cell membrane. The proposed empirical relationship between fold change in the OAT1/3 CL_int_,_u,active_ and *f*_u,inc_ enables the initial prospective evaluation of the extent of the albumin-mediated uptake effect for other OAT1/3 substrates and facilitates the selection of compounds for subsequent albumin-mediated uptake studies. Substantial improvement to the IVIVE of OAT1/3-mediated CL_sec_ and CL_r_ was observed using measurements performed in the presence of albumin combined with the derived REF and RAF scalers. Based on the current findings, we recommend the inclusion of HSA in *in vitro* OAT1/3 assays to improve the accuracy of the bottom-up prediction of OAT1/3-mediated CL_sec_ and CL_r_ during drug development.

## Supporting Information Available

Unbound substrate concentration used in the OAT1/3 assay (Table S1), detailed LC-MS/MS parameters (Table S2), estimated parameters of the facilitated-dissociation model using active or active plus passive CL_int,u_ (Table S3), estimated parameters of the reduced facilitated-dissociation model (Table S4), predicted versus observed *in vivo* CL_int,sec_, CL_sec_, and CL_r_ based on measurements in the presence and absence of human serum albumin (Table S5), impact of the albumin-mediated uptake effect on the predictive performance of *in vivo* CL_int,sec_, CL_sec_ and CL_r_ (Table S6), effect of varying numbers of proximal tubular cells per gram of kidney cortex (PTCPGK) on predicted renal clearance (Table S7), nonspecific binding of OAT1/3 substrates (Table S8), *f*_u,inc_ of 4-pyridoxic acid in the presence of 0.5 to 4.0% HSA (Table S9), measured *f*_u,inc_ in the presence of 4% HSA versus the observed *f*_u,p_ (Figure S1), intracellular uptake of adefovir with ciPTEC-OAT3 and rivaroxaban, rosuvastatin, and oseltamivir carboxylate with ciPTEC-OAT1 (Figure S2), intracellular uptake of furosemide with ciPTEC-OAT1 (Figure S3), comparison of the predicted fraction eliminated by OAT1 and OAT3 and glomerular filtration among total renal clearance (Figure S4), uptake of 4-pyridoxic acid (PDA) versus time profile when total or unbound concentration were kept constant (Figure S5), investigation of 4-pyridoxic acid (PDA) passive diffusion with inclusion of 1% HSA (Figure S6), preliminary optimization of the albumin-mediated uptake effect assay, and OAT1/3 proteomic quantification methodology (PDF)

## Abbreviations

*B*_max_: maximum binding capacity of albumin with the surface of the cell membrane
ciPTEC: conditionally immortalized proximal tubular epithelial cells
CL_int,sec_: intrinsic secretion clearance
CL_int,u_: unbound intrinsic clearance
CL_int,u,active_: unbound active intrinsic clearance
CL_int,u,passive_: unbound passive diffusion clearance
CL_r_: renal clearance
CL_sec_: renal secretion clearance
EFD: extent of facilitated dissociation
*F*_reabs_: fraction of drug reabsorbed from the kidney tubules back into systemic circulation
*f*_u,inc_: fraction unbound in the incubation
*f*_u,b_: fraction unbound in blood
*f*_u,p_: fraction unbound in plasma
GFR: glomerular filtration rate
GMFE: geometric mean fold error
HSA: human serum albumin
IVIVE: *in vitro-*to-*in vivo* extrapolation
*K*_d_: dissociation constant of the ligand from albumin
*K*_d,m_: binding affinity of albumin for the surface of the cell membrane
LC-MS/MS: liquid chromatography-tandem mass spectrometry
OAT1/3: organic anion transporter 1/3
OATP1B1/3: organic anion transporting polypeptide 1B1/3
PTCPGK: proximal tubular cell per gram of kidney cortex
*Q*_r_: renal blood flow
RAF: relative activity factor
REF: relative expression factor
RMSE: root-mean-square error
*V*_b,max_: clearance capacity for a ligand transported via the facilitated-dissociation mechanism
