# Supplementary material for: Albumin-Mediated Drug Uptake by Organic Anion Transporter 1/3 Is Real: Implications for the Prediction of Active Renal Secretion Clearance
Source: Mol Pharm. 2024 Aug 21;21(9):4603–17. doi: 10.1021/acs.molpharmaceut.4c00504 (PMC11372837; doi:10.1021/acs.molpharmaceut.4c00504)
Supplement: Supplementary file 1 — mp4c00504_si_001.pdf [file mp4c00504_si_001.pdf]

Supplementary Information

Albumin-Mediated Drug Uptake by Organic Anion Transporter 1/3 is Real: Implications for Prediction of Active Renal Secretion Clearance

Shawn Pei Feng Tan, Annika Tillman, Susan J. Murby, Amin Rostami-Hodjegan, Daniel Scotcher, and Aleksandra Galetin

Table S1. Unbound substrate concentration used in the OAT1/3 uptake assay and the reported Michaelis-Menten constant (K_m) value for each investigated substrate for the OAT1 and OAT3.

Substrate	Reported K_m (μM)		Unbound Concentration in Assay (μM)	
	OAT1	OAT3	OAT1	OAT3
Olmesartan	0.0683[1]	0.12[1]	0.050	0.050
Bumetanide	NR	1.59[2]	1.0	1.0
Furosemide	38.9[3]	21.5[3]	NA	4.0
Rivaroxaban	NA[4]	8.91[4]	NA	0.2
4-Pyridoxic Acid	33[5]	52.1[5]	1.0	5.0
Rosuvastatin	NA[6]	7.3[6]	NA	1.0
Oseltamivir Carboxylate	NA[7]	NR	NA	50
Adefovir	23.8[8]	NA[7,9]	2.0	NA

NA, Not applicable as not a substrate for that transporter.
NR, Not reported.

Table S2. Detailed LC-MS/MS parameters

**Column**	Luna C18 (3 μm, 50 × 4.6 mm) column
**Flow Rate**	1.0 mL/min, split to 0.25 mL/min before delivery into MS
**Mobile Phase**	A: 90% water, 10% methanol, and 0.05% formic acid<br>B: 10% water, 90% methanol, and 0.05% formic acid<br>C: 90% water, 10% methanol, and 1 mM ammonium acetate<br>D: 10% water, 90% methanol, and 1 mM ammonium acetate

**Analyte**	**ESI**	**Internal Standard**	**Gradient Condition** Time (min)	Mobile Phase (%) A	B	C	D	**Q1 m/z**	**Q3 m/z**
Adefovir	Negative	Gemfibrozil	0	100	-	-	-	271.95	133.95
			2	100	-	-	-		
			3	-	80	20	-		
			4	-	-	-	100		
			5	100	-	-	-		
			5.5	100	-	-	-		
Furosemide	Negative	Gemfibrozil	0	-	-	100	-	328.95	285.10
			1	-	-	100	-		
			3	-	100	-	-		
			4	-	-	-	100		
			5	-	-	100	-		
			5.5	-	-	100	-		
Oseltamivir Carboxylate	Negative	Gemfibrozil	0	-	-	100	-	283.05	195.00
			1	-	-	100	-		
			3	-	100	-	-		
			4	-	-	-	100		
			5	-	-	100	-		
4-Pyridoxic Acid	Negative	Gemfibrozil	0	-	-	100	-	181.80	137.95
			1	-	-	100	-		
			3	-	50	-	50		
			4	-	50	-	50		
			5	-	-	100	-		
Bumetanide	Positive	Buspirone	0	100	-	-	-	365.05	240.20
			1	100	-	-	-		
			3	-	80	20	-		
			4	-	-	-	100		
			5	100	-	-	-		
Olmesartan	Positive	Buspirone	0	100	-	-	-	447.05	207.05
			1	100	-	-	-		
			3	5	85	10	-		
			4	-	100	-	-		
			5	100	-	-	-		
Rivaroxaban	Positive	Buspirone						457.95	414.15

			0 100 - - -<br>1 100 - - -<br>3 - 80 20 -<br>4 - - - 100<br>5 100 - - -		
Rosuvastatin	Positive	Buspirone	Time (min) / Mobile Phase (%): A B C D<br>0 100 - - -<br>1 100 - - -<br>3 5 85 10 -<br>4 - 90 5 5<br>5 100 - - -	482.15	258.25
Gemfibrozil	Negative	-	-	249.25	121.00
Buspirone	Positive	-	-	386.15	122.00

**Table S3.** Estimated parameters when fitting the facilitated-dissociation model to measured $CL_{int,u}$ (OAT1/3 active uptake plus passive diffusion) or $CL_{int,u,active}$ (OAT1/3 active uptake only)[a]				
	**Active Uptake Only**		**Active and Passive Uptake**	
	**$V_{b,max}$[b] (pmol/min/mg)**			
**Substrate**	**OAT1**	**OAT3**	**OAT1**	**OAT3**
Olmesartan	72.7 ± 15.0	44.2 ± 12.3	75.7 ± 15.5	46.2 ± 12.3
Bumetanide	447 ± 48	470 ± 132	467 ± 49.4	492 ± 144
4-Pyridoxic Acid	213 ± 24	51.9 ± 15.8	222 ± 24.6	53.1 ± 17.1
Adefovir	3055 ± 188000[c]	-	-1083 ± 194000[c]	-
Furosemide	-	453 ± 127	-	478 ± 139
Rivaroxaban	-	714 ± 201	-	723 ± 216
Rosuvastatin	-	281 ± 96	-	290 ± 101
Oseltamivir Carboxylate	-	-1120 ± 2640[c]	-	-1150 ± 2660[c]
	**$K_{d,m}$[b] (μM)**			
	542 ± 217		581 ± 243	

[a] Data presented as mean ± S.D.
[b] Estimated by fitting Equation 4 to measured $CL_{int,u,active}$ and albumin dissociation constant (K_d).
[c] Uncertainty in $V_{b,max}$ estimates because these are negative controls for the albumin-mediated uptake effect that are not protein-bound and have a large K_d.

**Table S4.** Estimated parameters of the reduced facilitated-dissociation model[a]			
**EFD (μM)**	**ciPTEC-OAT1**[b]	**ciPTEC-OAT3**[b]	**Hepatocytes-OATP1B1/3**[c]
	641 ± 137	513 ± 265	11, 5.2
**$K_{d,m}$ (μM)**	520 ± 434		46

[a] Data presented as mean ± S.D.
[b] Estimated by fitting the reduced facilitated-dissociation model (Equation 6) to measured ratio of $CL_{int,u,active}$ (HSA/control) and K_d.
[c] Published estimates obtained from hepatocyte experiments with organic anion transporting polypeptide 1B1/3 substrates[10], data presented as mean.

**Table S5.** Predicted versus observed *in vivo* intrinsic secretion clearance ($CL_{int,sec}$), secretion clearance (CL_{sec}) and renal clearance (CL_r) based on measurements in the presence and absence of human serum albumin (HSA)[a]												
**Substrates**	**$CL_{int,sec}$ (L/h)**				**CL_{sec} (L/h)**				**CL_r (L/h)**			
	**Predicted**			**Observed**	**Predicted**			**Observed**	**Predicted**			**Observed**
	**0% HSA**	**1% HSA**	**4% HSA**		**0% HSA**	**1% HSA**	**4% HSA**		**0% HSA**	**1% HSA**	**4% HSA**	
**Relative Expression Factor Approach**												
Adefovir	42.9	57.8	44.7	10.8	21.3	25.1	21.8	7.82	28.3	32.0	28.8	14.2
Bumetanide	11.2	69.2	167	680	0.20	1.23	2.88	5.51	0.33	1.36	3.01	5.77
Furosemide	5.83	37.8	115	190	0.19	1.17	3.32	5.07	0.42	1.40	3.55	5.31
Olmesartan	7.92	85.2	284	228	0.02	0.22	0.74	0.59	0.04	0.24	0.75	0.61
Oseltamivir Carboxylate	2.33	2.28	2.12	11.7	1.92	1.88	1.76	8.29	8.91	8.87	8.74	15.6
4-Pyridoxic Acid	3.46	7.85	12.6	340	0.29	0.66	1.05	14.8	0.92	1.28	1.67	15.6
Rivaroxaban	6.78	41.7	67.0	41.2	0.48	2.79	4.32	2.63	1.00	3.31	4.84	3.17
Rosuvastatin	6.80	13.6	17.7	130	0.78	1.52	1.96	11.1	1.63	2.37	2.81	11.9
**Relative Activity Factor Approach[a]**												
Bumetanide	52.5	299	688	680	0.94	4.98	10.3	5.51	1.07	5.11	10.5	5.77
Furosemide	29.4	191	579	190	0.91	5.20	12.2	5.07	1.14	5.43	12.4	5.31
Olmesartan	34.2	355	976	229	0.09	0.92	2.40	0.59	0.11	0.93	2.42	0.61
4-Pyridoxic Acid	8.79	22.1	32.6	340	0.74	1.79	2.57	14.8	1.36	2.41	3.20	15.6
Rivaroxaban	34.1	210	337.7	41.2	2.32	11.1	15.4	2.63	2.84	11.7	16.0	3.17
Rosuvastatin	34.3	68.3	89.0	130	3.64	6.66	8.27	11.1	4.49	7.51	9.12	11.9
[a] Predictions for adefovir and oseltamivir carboxylate were not compared against clinical data with the relative activity factor (RAF) approach as clinical measurements of CL_r were used to derive the RAF scaler.												

**Table S6.** The impact of albumin-mediated uptake effect on the predictive performance of *in vivo* intrinsic secretion clearance ($CL_{int,sec}$), secretion clearance (CL_{sec}) and renal clearance (CL_r)									
	**Number of Substrates within 2-Fold Error**			**Number of Substrates within 3-Fold Error**			**Geometric Mean Fold Error**		
	**0% HSA**	**1% HSA**	**4% HSA**	**0% HSA**	**1% HSA**	**4% HSA**	**0% HSA**	**1% HSA**	**4% HSA**
**Relative Expression Factor Approach**									
$CL_{int,sec}$	0/8	1/8	3/8	0/8	2/8	3/8	18.4	5.9	4.0
CL_{sec}	0/8	1/8	4/8	1/8	2/8	5/8	13.3	4.3	3.0
CL_r	2/8	2/8	5/8	2/8	4/8	6/8	6.8	3.2	2.3
**Relative Activity Factor Approach[a]**									
$CL_{int,sec}$	1/6	3/6	2/6	1/6	4/6	2/6	6.8	2.8	3.4
CL_{sec}	1/6	4/6	2/6	1/6	4/6	3/6	5.0	2.2	3.1
CL_r	1/6	4/6	2/6	2/6	4/6	3/6	4.1	2.0	2.9

[a] Predictions for adefovir and oseltamivir carboxylate were not compared against clinical data with the relative activity factor (RAF) approach as clinical measurements of CL_r were used to derive the RAF scaler.

**Table S7.** Effect of varying number of proximal tubular cells per gram of kidney cortex (PTCPGK) on predicted renal clearance (CL_r) using the relative expression factor approach									
	**Number of Substrates within 2-Fold Error**			**Number of Substrates within 3-Fold Error**			**Geometric Mean Fold Error**		
**PTCPGK (PTC/g)**	**0% HSA**	**1% HSA**	**4% HSA**	**0% HSA**	**1% HSA**	**4% HSA**	**0% HSA**	**1% HSA**	**4% HSA**
99.4×10^6	2/8	2/8	5/8	2/8	4/8	6/8	6.8	3.2	2.3
209×10^6	1/8	3/8	3/8	3/8	6/8	7/8	5.0	2.4	2.2
60×10^6	2/8	3/8	4/8	2/8	3/8	5/8	7.8	4.1	2.6

**Table S8.** Non-specific binding of OAT1/3 substrates performed using uptake buffer containing human serum albumin (HSA) obtained from the OAT1/3 uptake transporter experiments			
**Substrate**	$\mathbf{f_{u,nsb}}$		
	**0% HSA**	**1% HSA**	**4% HSA**
Bumetanide	0.89	1.05	0.99
Olmesartan	1.13	0.94	0.93
4-Pyridoxic Acid	0.85	0.96	0.85
Furosemide	1.07	1.05	1.00
Rosuvastatin	0.93	0.98	0.93
Rivaroxaban	0.95	0.92	0.99
Adefovir	1.06	1.02	1.13
Oseltamivir Carboxylate	1.07	1.12	1.15

$f_{u,nsb}$: fraction unbound due to non-specific binding to plastic on 24-wellplates, calculated as the ratio of measured versus nominal substrate concentration in the uptake buffer.

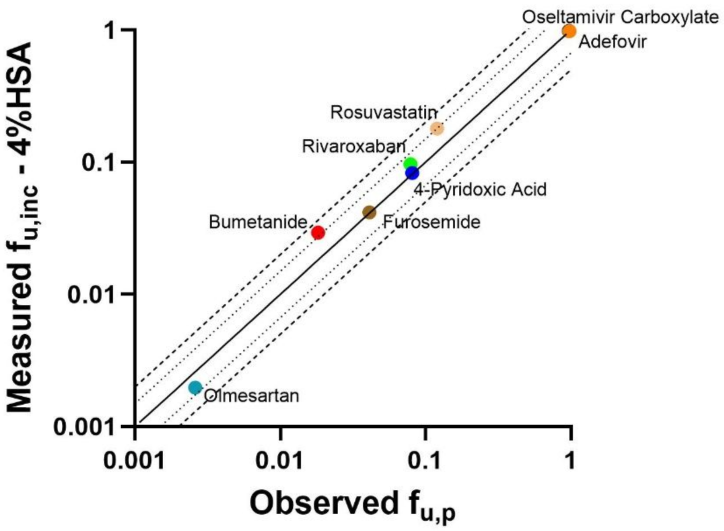

Figure S1. Measured fraction unbound in the incubation media ($f_{u,inc}$) in the presence of 4% HSA versus the observed fraction unbound in plasma ($f_{u,p}$). Solid, dotted, and dashed lines represent the line of unity, 1.5-fold error criterion and 2-fold error criterion respectively.

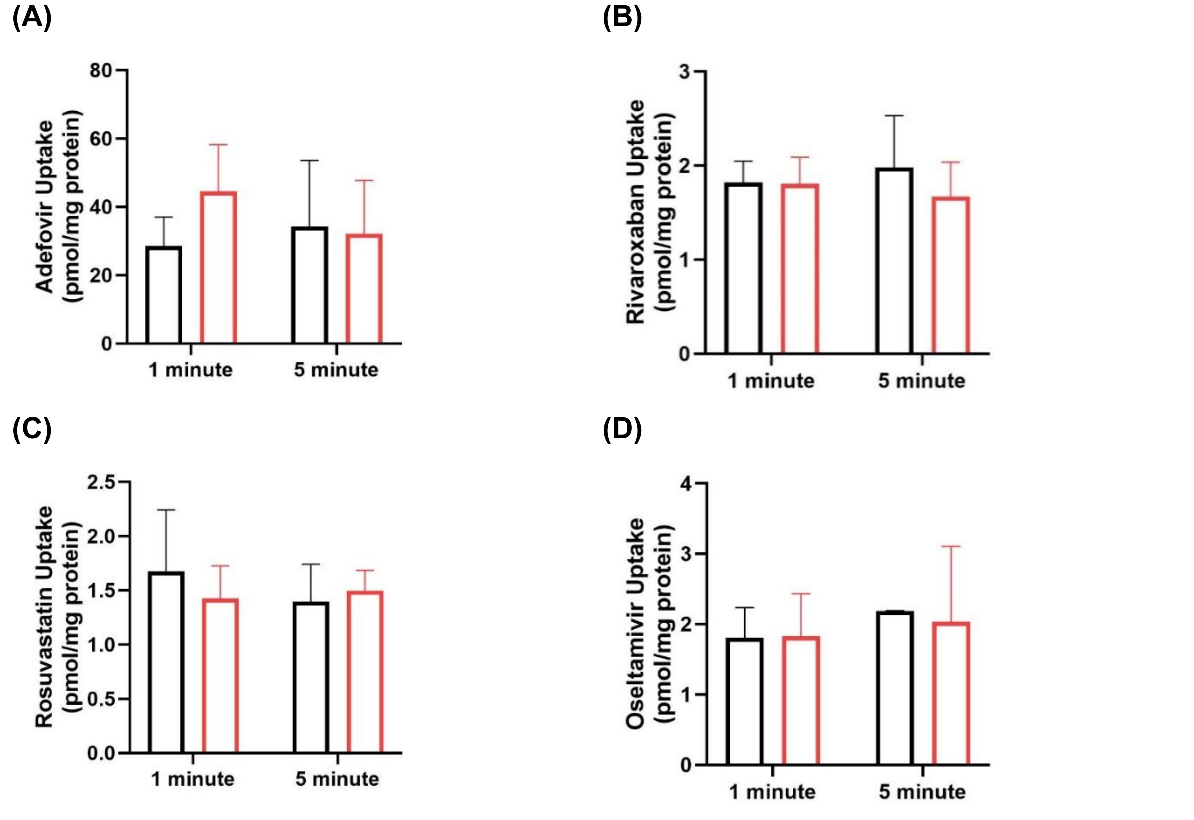

Figure S2. Intracellular uptake of adefovir **(A)** with ciPTEC-OAT3 and rivaroxaban **(B)**, rosuvastatin **(C)** and oseltamivir carboxylate **(D)** with ciPTEC-OAT1 in the control phase (black bars) and probenecid 300μM phase (red bars) after one and five minute incubation. Data represents mean ± SD of triplicates.

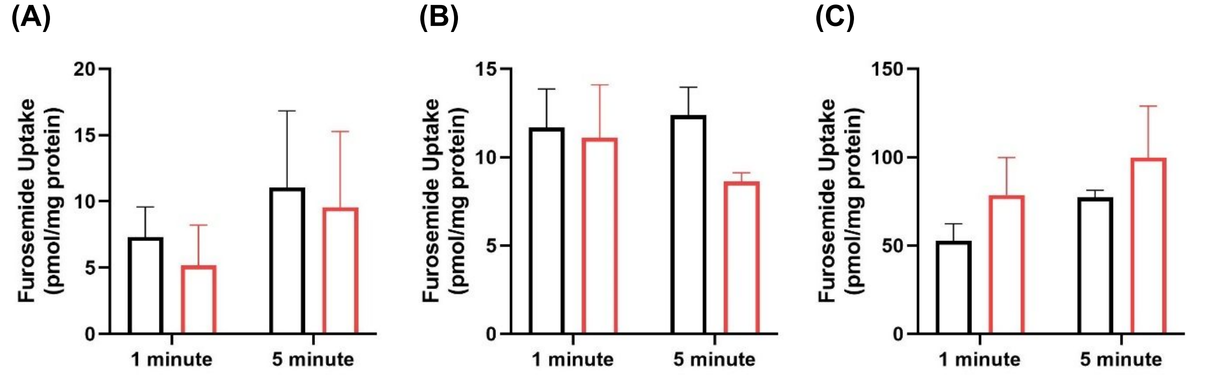

Figure S3. Intracellular uptake of furosemide with ciPTEC-OAT1 in the control phase (black bars) and probenecid 300μM phase (red bars). Furosemide 5.0μM (A), 10μM (B) and 50μM (C) were incubated for one and five minutes. Data represents mean ± SD of triplicates.

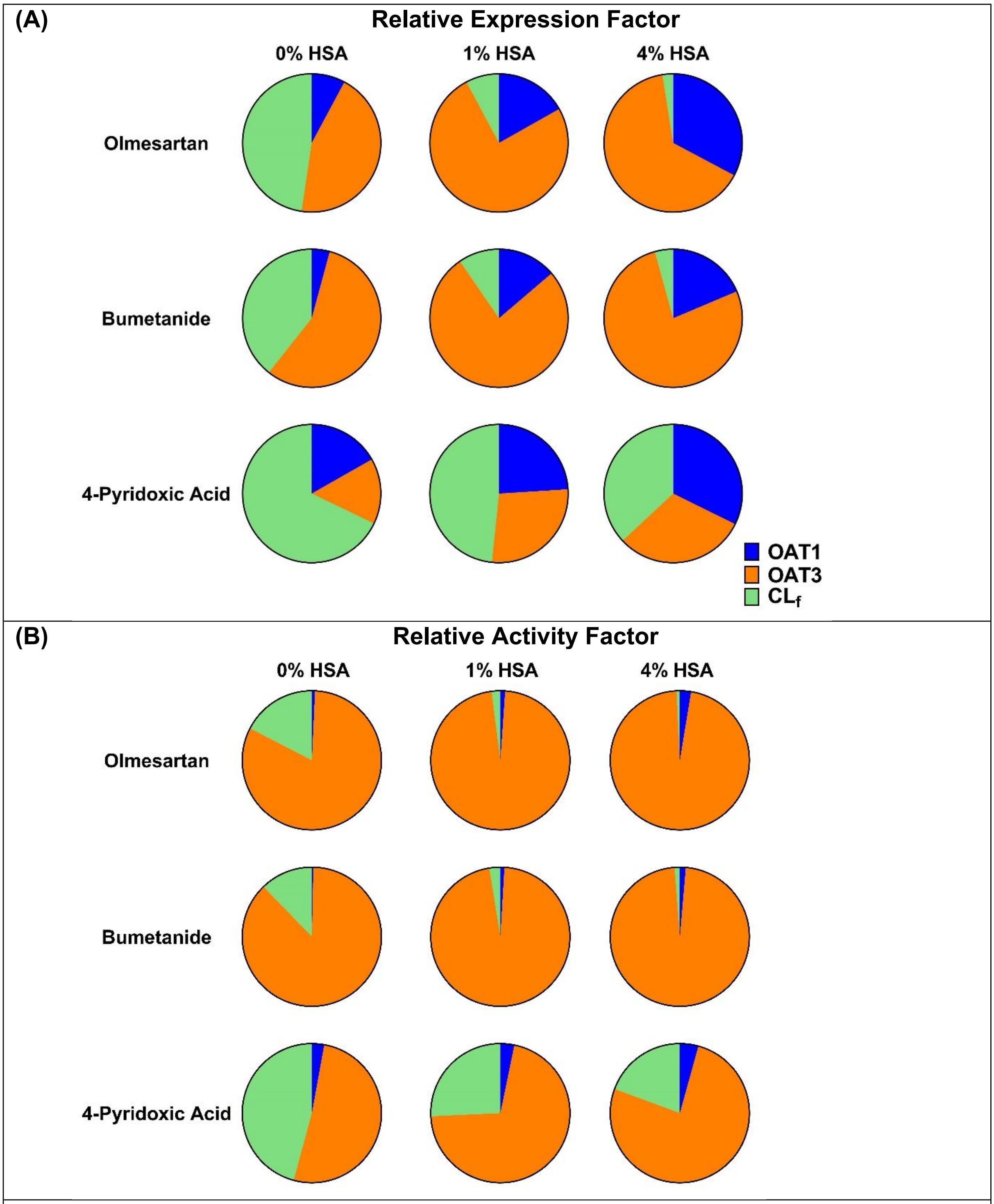

Figure S4. Comparison of predicted percentage eliminated by OAT1 (blue) and OAT3 (orange) renal secretion clearance, and glomerular filtration clearance (CL_f, green) among total renal clearance. The fraction eliminated by OAT1 and OAT3 renal secretion was predicted using the relative expression factor (A) or relative activity factor (B) approach with transporter kinetic measurements performed in the presence and absence of 1% and 4% human serum albumin.

Preliminary Optimisation of the Albumin-Mediated Uptake Effect Assay

Preliminary experiments were performed with 4-pyridoxic acid (PDA), an OAT1/3 endogenous biomarker, to optimise the experimental design of the albumin-mediated uptake assay. The optimisation includes deciding on the concentration of HSA used in the assay and whether the concentration of substrate should vary with the amount of HSA used. Once optimised, subsequent experiments with the shortlisted OAT1/3 substrates were performed. The OAT1-mediated uptake of PDA was investigated in the presence of 0% (control), 0.5%, 1.0%, 2.0% and 4.0% HSA in the substrate containing uptake buffer. Measurements of fraction unbound in the incubation buffer ($f_{u,inc}$) was performed using equilibrium dialysis for each experimental condition between 0 – 4.0% HSA **(Table S9)**. The concentration of PDA was either kept constant at a total concentration of 10 µM, or the unbound concentration was kept constant at 1.0 µM by accounting for the $f_{u,inc}$ in the presence of 0.5 – 4.0% HSA. When total concentration of PDA was kept constant at 10 µM, the unbound concentration of PDA would decrease proportionally to the change in $f_{u,inc}$ as increasing amounts of HSA was included in the assay.

When keeping the total concentration of PDA constant at 10 µM, the total PDA uptake versus time decreased with increasing HSA concentration **(Figure S5A)**. This trend can be rationalised by the decreasing unbound PDA concentration and $f_{u,inc}$ **(Table S9)**. But, when the OAT1-mediated intrinsic clearance ($CL_{int,active}$) was normalised against the measured $f_{u,inc}$, the unbound $CL_{int,active}$ ($CL_{int,u,active}$) increased with the inclusion of HSA **(Figure S5C)**. Compared against the control, $CL_{int,u,active}$ increased by 1.50, 1.82, 0.93 and 2.2-fold in the presence of 0.5%, 1.0%, 2.0% and 4.0% HSA. Similar effects were observed when unbound concentration of PDA was kept constant at 1.0 µM. By keeping the unbound concentration of PDA constant, the total PDA concentration in the assay and the corresponding uptake increased as larger amounts of HSA was added **(Figure S5B)**. At 0.5%, 1.0%, 2.0% and 4.0% HSA, $CL_{int,u,active}$ increased by 1.50, 1.92, 1.79 and 3.46-fold when compared against the control **(Figure S5D)**. In conclusion, keeping the total or unbound substrate concentration constant between the various HSA concentrations had a similar trend of fold increase in the $CL_{int,u,active}$. As keeping the unbound substrate concentration constant benefited from improved analytical sensitivity versus keeping total substrate concentration constant (where unbound concentrations would decrease by several magnitudes at 4% HSA), further experiments with the shortlisted OAT1/3 substrates were performed by keeping unbound concentration constant between control and HSA condition.

Additional preliminary experiments were performed with PDA to understand if passive diffusion changes with the inclusion of HSA. OAT1-mediated uptake of PDA was investigated in the presence of 0% (control), 300 µM probenecid, 1.0% HSA and 1.0% HSA with 300 µM probenecid. Once again, the unbound concentration of PDA was kept constant at 1.0 µM across these conditions by measuring the $f_{u,inc}$. As probenecid binds to HSA as well (observed fraction unbound in plasma = 0.10), $f_{u,inc}$ was measured in the presence of 1.0% HSA and unbound concentration kept constant at 300 µM. Our results show minimal changes to the unbound passive diffusion clearance (CL_{pd}) of PDA upon the inclusion of 1.0% HSA. Uptake of PDA was similar between 300 µM probenecid and 1.0% HSA + 300 µM probenecid condition **(Figure S6)** and PDA CL_{pd} was 0.031 µL/min/mg and 0.039 µL/min/mg, respectively.

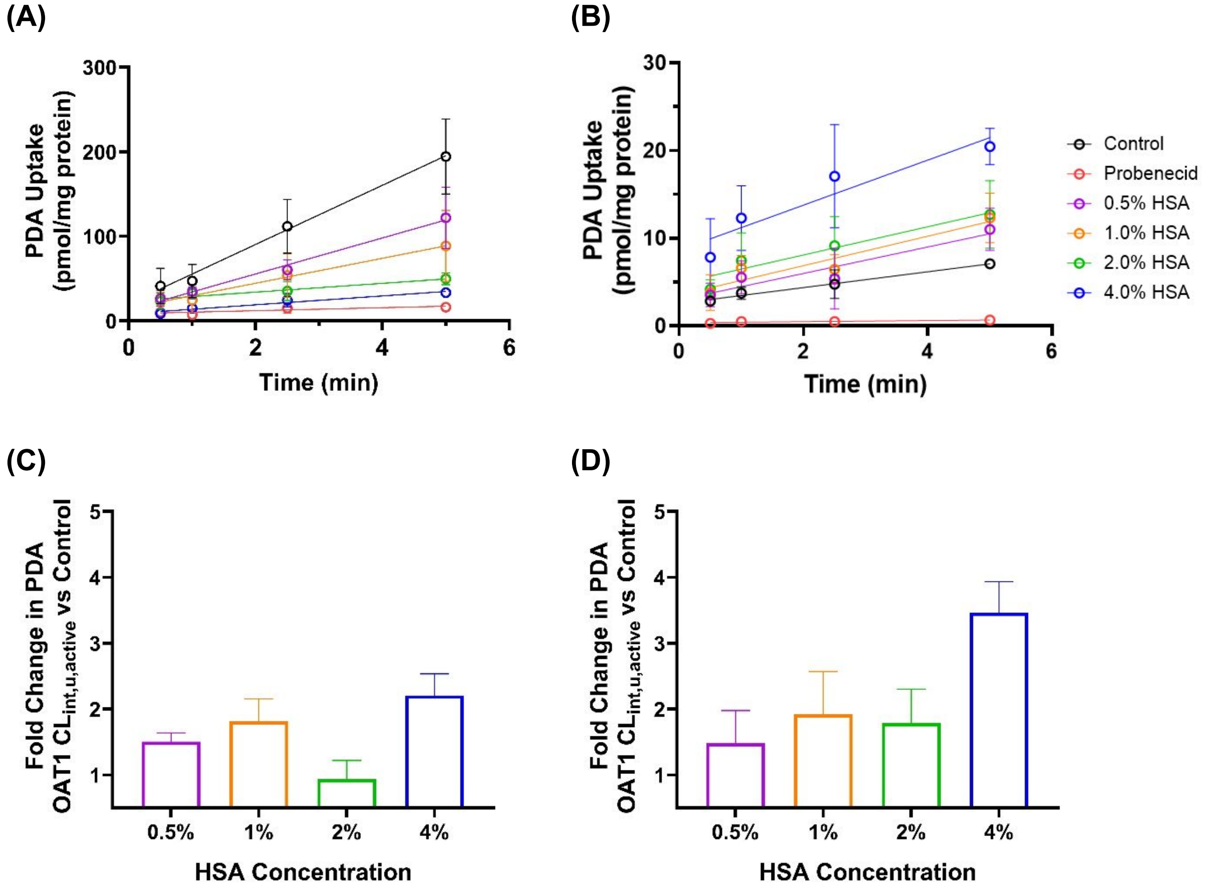

Figure S5. Uptake of 4-pyridoxic acid (PDA) versus time profile **(A, B)** and the fold change in OAT1 $CL_{int,u,active}$ versus the control (HSA/control) **(C, D)** in the presence of 0.5 to 4% HSA. Two experimental designs were used when investigating the OAT1-mediated uptake of PDA, keeping either total PDA concentration constant at 10 μM **(A, C)** or unbound PDA concentration constant at 1.0 μM **(B, D)** with increasing HSA concentrations. The open circles **(A,B)** and bars **(C,D)** represent the mean uptake of PDA into the ciPTEC-OAT1 and fold-change in $CL_{int,u,active}$ versus control, respectively. Error bars represent the standard deviation. Data represent mean ± SD of 3 to 4 independent experiments.

Table S9. Fraction unbound in the incubation system ($f_{u,inc}$) of 4-pyridoxic acid in the presence of 0.5 to 4.0% human serum albumin (HSA).

	**0.5% HSA**	**1% HSA**	**2% HSA**	**4% HSA**
$f_{u,inc}$	0.400 ± 0.042	0.298 ± 0.005	0.163 ± 0.005	0.079 ±0.006

Data presented as mean ± S.D.

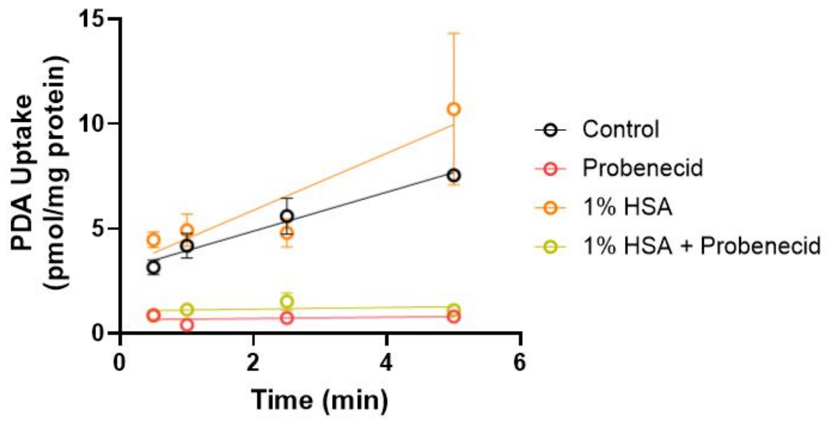

Figure S6. Uptake of 4-pyridoxic acid (PDA) versus time profile in the presence of 0% (control), 300 μM probenecid, 1.0% HSA and 1.0% HSA with 300 μM probenecid. Unbound concentration of PDA and probenecid was kept constant at 1.0 μM and 300 μM across these conditions by measuring the $f_{u,inc}$. The open circles and error bars represent the mean ± standard deviation of triplicate data.

OAT1/3 Proteomic Quantification Methodology

Briefly, total membrane protein was isolated from conditionally immortalised proximal tubular epithelial cells overexpressing OAT1 or OAT3 cultured on 24-wellplates using the Calbiochem ProteoExtract® Native Membrane Protein Extraction Kit (Sigma-Aldrich, Dorset, UK) according to the manufacturer's protocol. Subsequently, 35 µg of membrane protein was incubated with sodium deoxycholate (5% w/v) and dithiothreitol (0.1 M) for 10 minutes at room temperature then 30 minutes at 56°C to denature and reduce the membrane proteins. This is followed by alkylation with iodoacetamide (50 mM) for 30 minutes in the dark. The proteins were digested for 3 hours at 30°C using LysC (1:20 ratio of enzyme to protein). Trypsin (1:25 ratio of enzyme to protein) was then added for a 16-hour long digestion at 37°C. The resulting peptides were desalted and washed using a C18 spin-column. After elution, the samples were vacuum-dried and stored at -80°C before analysis. Absolute protein quantification was performed by untargeted LC-MS/MS on a Q ExactiveTM HF Hybrid Quadrupole-Orbitrap™ (ThermoFisher Scientific, Bremen, Germany), as described previously.[11] Abundance of OAT1 and OAT3 (N = 4 replicates each) was quantified using the total protein approach.[12]

References

(1) Yamada, A.; Maeda, K.; Kamiyama, E.; Sugiyama, D.; Kondo, T.; Shiroyanagi, Y.; Nakazawa, H.; Okano, T.; Adachi, M.; Schuetz, J. D.; et al. Multiple human isoforms of drug transporters contribute to the hepatic and renal transport of olmesartan, a selective antagonist of the angiotensin II AT1-receptor. *Drug Metab Dispos* **2007**, *35* (12), 2166-2176.

(2) Hasannejad, H.; Takeda, M.; Taki, K.; Shin, H. J.; Babu, E.; Jutabha, P.; Khamdang, S.; Aleboyeh, M.; Onozato, M. L.; Tojo, A.; et al. Interactions of human organic anion transporters with diuretics. *J Pharmacol Exp Ther* **2004**, *308* (3), 1021-1029.

(3) Ebner, T.; Ishiguro, N.; Taub, M. E. The Use of Transporter Probe Drug Cocktails for the Assessment of Transporter-Based Drug-Drug Interactions in a Clinical Setting-Proposal of a Four Component Transporter Cocktail. *J Pharm Sci* **2015**, *104* (9), 3220-3228.

(4) Cheong, E. J. Y.; Ng, D. Z. W.; Chin, S. Y.; Wang, Z.; Chan, E. C. Y. Application of a physiologically based pharmacokinetic model of rivaroxaban to prospective simulations of drug-drug-disease interactions with protein kinase inhibitors in cancer-associated venous thromboembolism. *Br J Clin Pharmacol* **2022**, *88* (5), 2267-2283.

(5) Shen, H.; Nelson, D. M.; Oliveira, R. V.; Zhang, Y.; McNaney, C. A.; Gu, X.; Chen, W.; Su, C.; Reily, M. D.; Shipkova, P. A.; et al. Discovery and Validation of Pyridoxic Acid and Homovanillic Acid as Novel Endogenous Plasma Biomarkers of Organic Anion Transporter (OAT) 1 and OAT3 in Cynomolgus Monkeys. *Drug Metab Dispos* **2018**, *46* (2), 178-188.

(6) Windass, A. S.; Lowes, S.; Wang, Y.; Brown, C. D. The contribution of organic anion transporters OAT1 and OAT3 to the renal uptake of rosuvastatin. *J Pharmacol Exp Ther* **2007**, *322* (3), 1221-1227.

(7) Mathialagan, S.; Piotrowski, M. A.; Tess, D. A.; Feng, B.; Litchfield, J.; Varma, M. V. Quantitative Prediction of Human Renal Clearance and Drug-Drug Interactions of Organic Anion Transporter Substrates Using In Vitro Transport Data: A Relative Activity Factor Approach. *Drug Metab Dispos* **2017**, *45* (4), 409-417.

(8) Ho, E. S.; Lin, D. C.; Mendel, D. B.; Cihlar, T. Cytotoxicity of antiviral nucleotides adefovir and cidofovir is induced by the expression of human renal organic anion transporter 1. *J Am Soc Nephrol* **2000**, *11* (3), 383-393.

(9) Uwai, Y.; Ida, H.; Tsuji, Y.; Katsura, T.; Inui, K. Renal transport of adefovir, cidofovir, and tenofovir by SLC22A family members (hOAT1, hOAT3, and hOCT2). *Pharm Res* **2007**, *24* (4), 811-815.

(10) Miyauchi, S.; Kim, S. J.; Lee, W.; Sugiyama, Y. Consideration of albumin-mediated hepatic uptake for highly protein-bound anionic drugs: Bridging the gap of hepatic uptake clearance between in vitro and in vivo. *Pharmacol Ther* **2022**, *229*, 107938.

(11) Al-Majdoub, Z. M.; Scotcher, D.; Achour, B.; Barber, J.; Galetin, A.; Rostami-Hodjegan, A. Quantitative Proteomic Map of Enzymes and Transporters in the Human Kidney: Stepping Closer to Mechanistic Kidney Models to Define Local Kinetics. *Clin Pharmacol Ther* **2021**, *110* (5), 1389-1400.

(12) El-Khateeb, E.; Al-Majdoub, Z. M.; Rostami-Hodjegan, A.; Barber, J.; Achour, B. Proteomic Quantification of Changes in Abundance of Drug-Metabolizing Enzymes and Drug Transporters in Human Liver Cirrhosis: Different Methods, Similar Outcomes. *Drug Metab Dispos* **2021**, *49* (8), 610-618.
